# Recent advances in the application of isoindigo derivatives in materials chemistry

**DOI:** 10.3762/bjoc.17.111

**Published:** 2021-07-06

**Authors:** Andrei V Bogdanov, Vladimir F Mironov

**Affiliations:** 1 A.E. Arbuzov Institute of Organic and Physical Chemistry, FRC Kazan Scientific Center of RAS, 8 Arbuzov St., Kazan 420088, Russian Federation

**Keywords:** isoindigo, OFET, photoactive polymers, photovoltaics, solar cells

## Abstract

In this review, the data on the application of isoindigo derivatives in the chemistry of functional materials are analyzed and summarized. These bisheterocycles can be used in the creation of organic solar cells, sensors, lithium ion batteries as well as in OFET and OLED technologies. The potentials of the use of polymer structures based on isoindigo as photoactive component in the photoelectrochemical reduction of water, as matrix for MALDI spectrometry and in photothermal cancer therapy are also shown. Data published over the past 5 years, including works published at the beginning of 2021, are given.

## Introduction

**Scheme 1:**
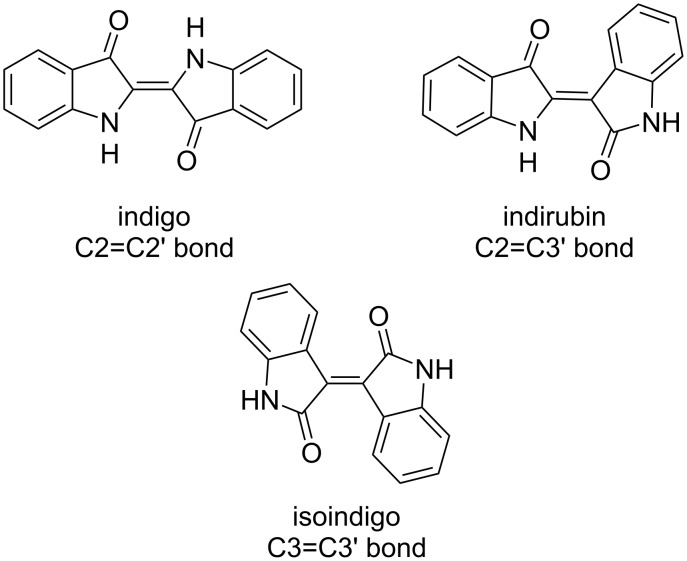
Representatives of isomeric bisoxindoles.

Among three isomeric bisoxindoles, isoindigo has recently attracted the greatest interest (Scheme 1). The first studies on this class of compounds were related to the field of medicinal chemistry since a number of isoindigo derivatives were found to be highly active against leukemia [1–3]. However, to date, the volume of publications on the study of the biological activity of isoindigo derivatives has been steadily decreasing.

At the same time, the unique properties of the isoindigo structure (planarity, stability, a high degree of conjugation, and electron deficiency) began to attract more and more attention of many research groups. In addition, the ease of modification, such as at the endocyclic nitrogen atom as aromatic fragment of isoindigo, makes it possible to fine-tune the electronic properties. These factors led to the beginning of many studies on isoindigo as a platform for the construction of polymeric materials for various purposes.

## Review

### Organic solar cells (OSCs) on the base of isoindigo derivatives

**Scheme 2:**
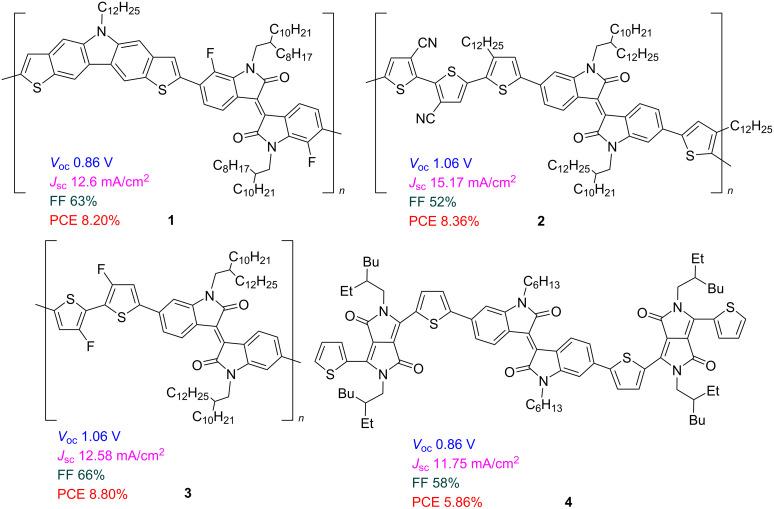
Isoindigo-based OSCs with the best efficiency.

Since the pioneering works on the use of isoindigo derivatives in the design of OSCs [3–5], specialists in this field have made significant progress in tuning and improving their properties [6–12]. The main photophysical characteristics that determine the effectiveness of OSCs are open circuit voltage (*V*_OC_), short-circuit current (*J*_SC_) and fill factor (FF). In addition, the solubility of isoindigo derivatives in organic solvents is very important since this affects the morphology of thin films of the photovoltaic cells. To date, the maximum efficiency of 12.05% has been shown by an OSC based on a composite of a donor polythiophene and an acceptor polymeric dicyanoindanone derivative [13]. Among the derivatives of isoindigo, the leading compounds are polymers **1**–**3**, which were used in the design of OSCs as donor components of the active layer. Their power conversion efficiency (PCE) reached more than 8%. The development of an OSC based on low-molecular-weight derivatives of type **4**, containing only one isoindigo fragment, also seems promising (Scheme 2).

**Scheme 3:**
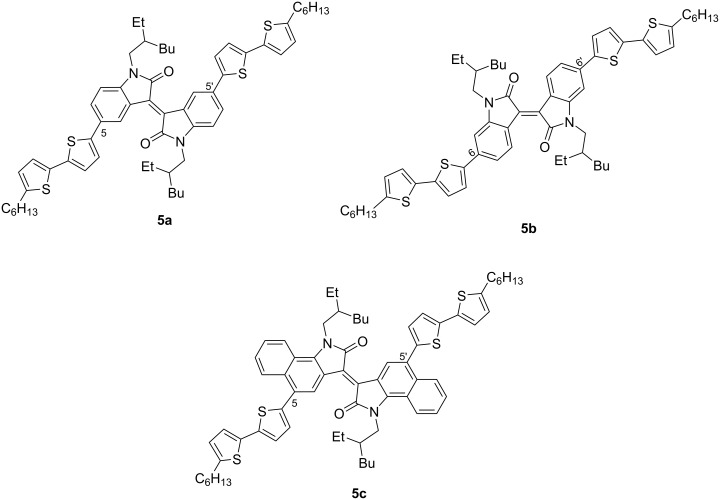
Monoisoindigos with preferred 6,6'-substitution.

One of the areas of research is the design of low-molecular-weight structures containing one or two isoindigo fragments in a unified conjugated electronic system. Currently, to improve the key characteristics of OSCs, some directions of studies are related to the design of substituents both on the heterocyclic platform (in position 1 and in the aromatic ring) and in the side chain. In the overwhelming majority of works, studies on the photophysical properties of isoindigo derivatives containing a thiophene fragment in position 6 are described. Thus, the authors of References [14–15] obtained a small number of simple representatives of symmetric dithiophene derivatives of isoindigos **5a**–**c** (Scheme 3). The constructed solar cells with an active layer based on a mixture of compounds **5** (donor) and PC_61_BM (acceptor) in a 1:1 ratio showed the dependence of the efficiency on the structure and position of substituents in the aromatic fragment of isoindigo. In the presented series, the system based on compound **5b** with an efficiency of 1.25% turned out to be the best.

**Scheme 4:**
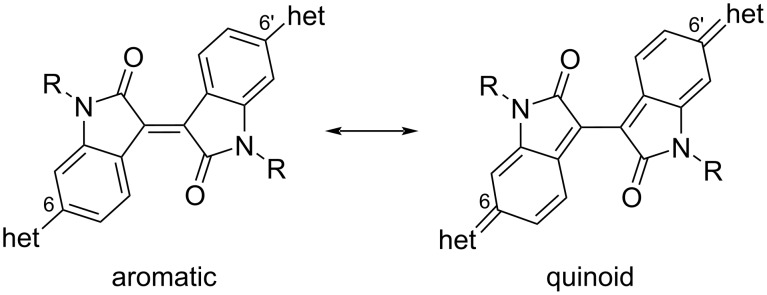
Possibility of aromatic–quinoid structural transition.

It was found [16] that 6,6'-substitution of the isoindigo core is preferable due to the possibility of the formation of a quinoid structure after irradiation with sunlight, which facilitates the transport of electrons through the system (Scheme 4).

**Scheme 5:**
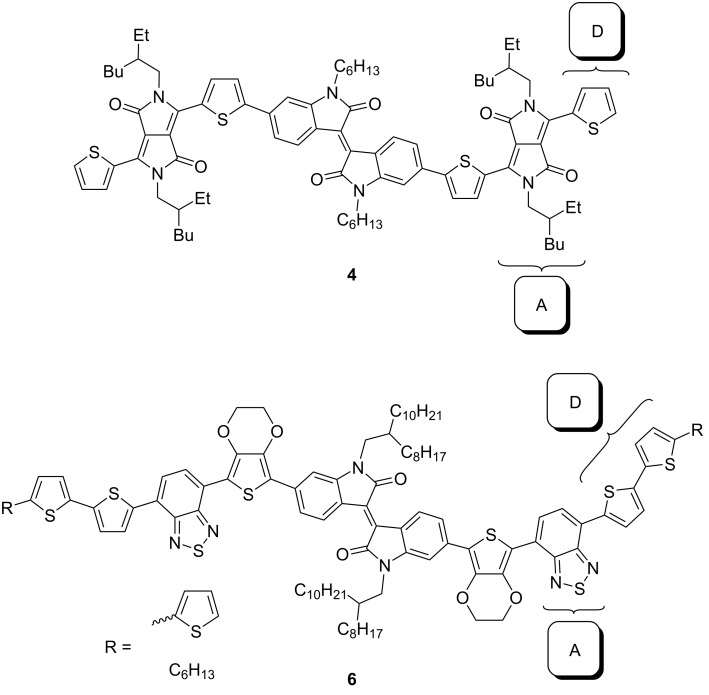
Isoindigo structures with incorporated acceptor nitrogen heterocycles.

Certain nitrogen heterocycles can be inserted into the substituent chain as an acceptor structural unit (Scheme 5). For example, an OSC based on a symmetrically substituted isoindigo derivative **4** containing a diketopyrrolopyrrole fragment in a mixture with PC_71_BM showed a record efficiency of 5.86% among oligomeric isoindigo [17]. At the same time, similarly constructed (D–A–D–A) oligomers **6** in the composition with PC_71_BM showed an efficiency of 1.3–1.4% [18].

**Scheme 6:**
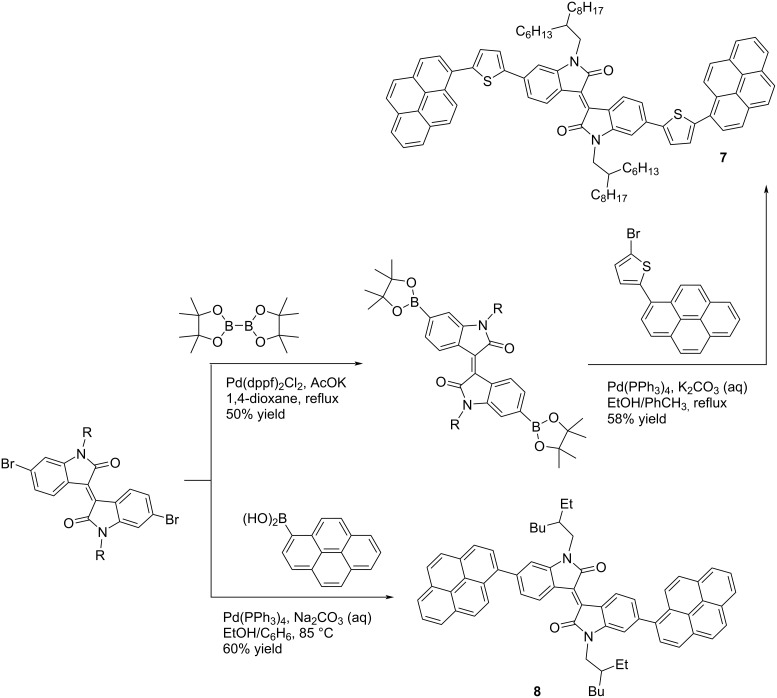
Monoisoindigos bearing pyrenyl substituents.

Another way to design isoindigoid OSCs is the introduction of aromatic substituents of variable nature into the oligomer structure. By the example of pyrene derivatives **7** and **8**, the dependence of the binding type of the aromatic fragment to the isoindigo core was revealed. The synthetic procedure for the preparation of these monoisoindigoid derivatives is based on the Suzuki reaction. At the same time, pyrene-1-ylboronic acid and 6,6'-dibromoisoindigo were used to introduce a pyrene fragment directly into the isoindigo nucleus, and to obtain a thiophene analogue, pyrenyl-substituted 2-bromothiophene and 6,6'-bis(4,4,5,5-tetramethyl-1,3,2-dioxaborolan-2-yl)isoindigo were used (Scheme 6). Thus, a photovoltaic cell based on thiophene derivative **7** mixed with PC_71_BM showed an efficiency of 1.88% [19], but if the pyrenyl substituent is bonded directly to the isoindigo core, the efficiency of such an OSC turns out to be significantly lower (0.10%) [20].

**Scheme 7:**
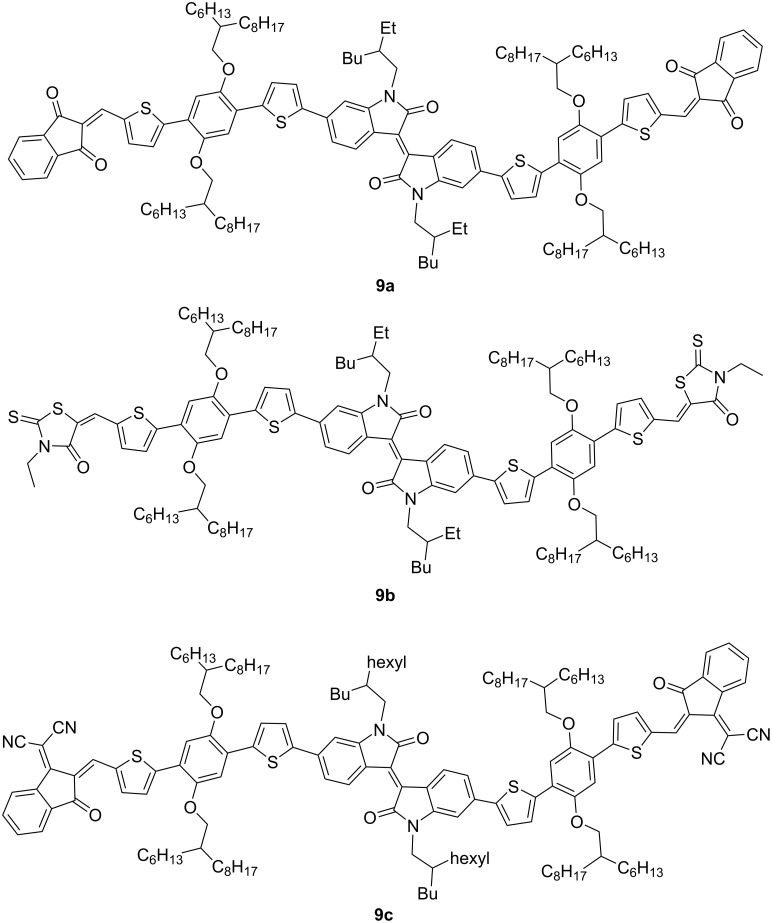
*p*-Alkoxyphenylene-embedded thienylisoindigo with different acceptor anchor units.

One of the highest efficiency values (4.7%) among low-molecular-weight isoindigo derivatives was shown by a two-component OSC based on isoindigo **9a**, containing an alkoxylated *p*-phenylene fragment and PC_71_BM in a ratio of 1:0.7 (w/w), a thin film of which was obtained from chloroform with 0.5 vol % *N*-methylpyrrolidone (Scheme 7). As the authors believe [21], the addition of this viscous solvent made it possible to provide a better surface morphology of a thin-film layer since without it, the efficiency was almost two times lower (2.8%). Therein, the key role of the structure of the acceptor terminal substituent was also revealed since a similar OSC based on the rhodamine derivative **9b** showed an efficiency of only 0.66% (Table 1).

**Table 1:** Processing conditions and additive influence on OSC performance based on monoisoindigos **9a**–**c**.

compound	second component (w/w)	additive	PCE, %

**9a**	PC_71_BM (1:0.5)	none	2.13
**9a**	PC_71_BM (1:0.7)	none	2.85
**9a**	PC_71_BM (1:0.7)	NMP^a^	4.70
**9b**	PC_71_BM (1:0.7)	none	0.66
**9b**	PC_71_BM (1:0.7)	NMP	—^b^
**9c**	J61 (1.3:1)	DPE^c^	2.82

^a^*N*-Methylpyrrolidone. ^b^Not possible to measure. ^c^Diphenyl ether.

It is important to note that the replacement of the thienylphenylene spacer in structure **9a** by the acceptor indan-3-dicyanoethylidene-1-one-2-ylidene fragment in compound **9c** led to a decrease in the efficiency to 2.82% [22].

**Scheme 8:**
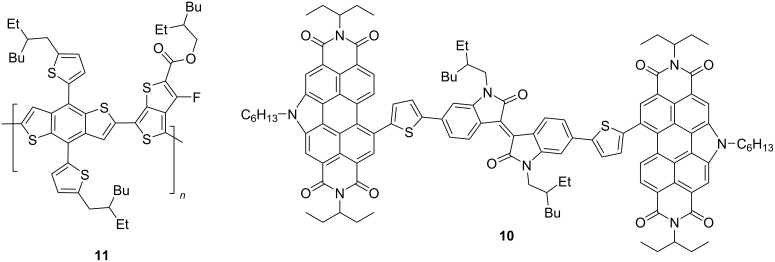
Nonfullerene OSC based on perylene diimide-derived isoindigo.

Perylene diimide-derived isoindigo derivative **10** was used as an acceptor in the creation of a nonfullerene OSC with thiophene polymer **11** as a donor component (Scheme 8). The PCE value of such a device turned out to be 2.6%.

**Scheme 9:**
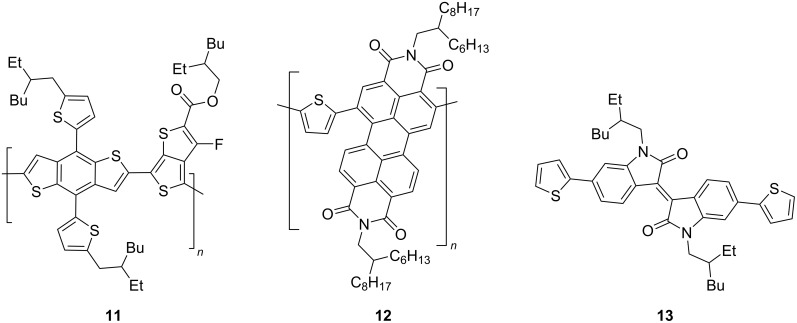
Isoindigo as an additive in all-polymer OSCs.

Cho et al. constructed a three-component cell in which the active layer consisted of a donor **11** and a polymeric acceptor based on perylene diimide **12** [23]. One of the simplest thiophene derivatives of isoindigo **13** was used here only as an additive (10 wt %), leading to an increase in efficiency from 5.9% to 6.8% (Scheme 9). Using a variety of physical methods, it has been proven that the presence of isoindigo **13** in the three-component mixture provides tighter packing of the thin layer and larger crystalline domains. This, in turn, leads to an increase in the decay time of the exciton and, as a consequence, to a high *J*_SC_ value.

**Scheme 10:**
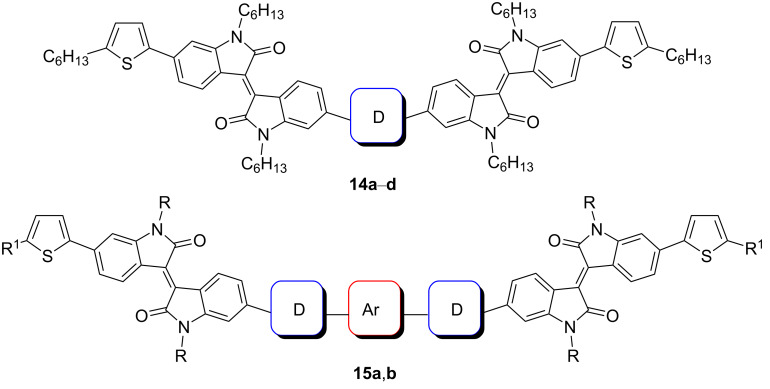
Bisisoindigos with different linker structures.

Several works have been devoted to the use of compounds containing two isoindigo units in the molecule for OSC creation. Scheme 10 shows examples of structures in which isoindigo fragments are linked either through a thiophene spacer **14** or through an embedded phenylene substituent **15**.

Thus, in the series of thiophene-centered bisisoindigos **14a**–**d**, the best efficiency values were shown by OSCs containing an odd number of thiophene units (**14a**: 2.16%; **14c**: 2.40%) [24]. A cell based on compound **15b** showed a close value of efficiency (2.25%) [25]. At the same time, incorporation of the tetrafluorophenylene fragment into the center of the molecule **14a** and the presence of a branched alkyl substituent at the nitrogen atom made it possible to improve the characteristics of the cell [26]. After annealing such an OSC at 80 °C, the efficiency was increased from 3 to 3.18%. It should be noted that in all cases mentioned here, PC_71_BM was used as the acceptor component of the OSC active layer. The photovoltaic characteristics of the OSCs based on compounds of this type are summarized in Table 2.

**Table 2:** Structures of linkers in bisisoindigos **14** and **15** and the performance of OSCs based on these.

compound	linker	*V*_OC_, V	*J*_SC_, mA/cm^2^	FF, %	PCE, %

**14a**	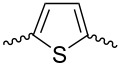	0.82	5.32	49.2	2.16
**14b**	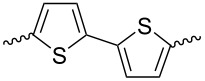	0.83	4.00	50.9	1.69
**14c**	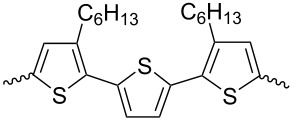	0.78	6.59	46.9	2.40
**14d**	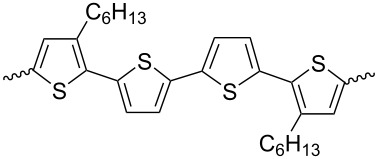	0.76	3.63	57.0	1.57
**15a**	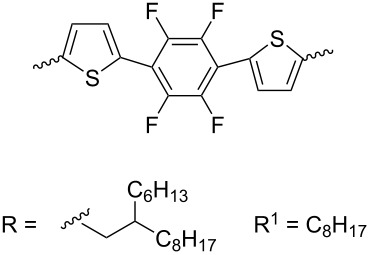	0.74	9.25	43.6	3.00
**15b**	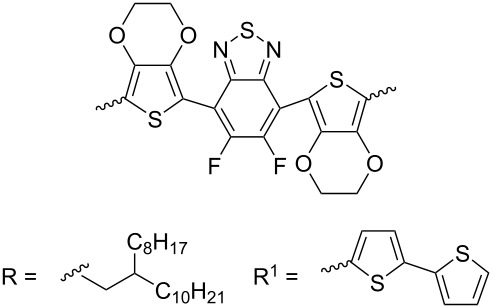	0.58	4.85	46.0	1.28

**Scheme 11:**
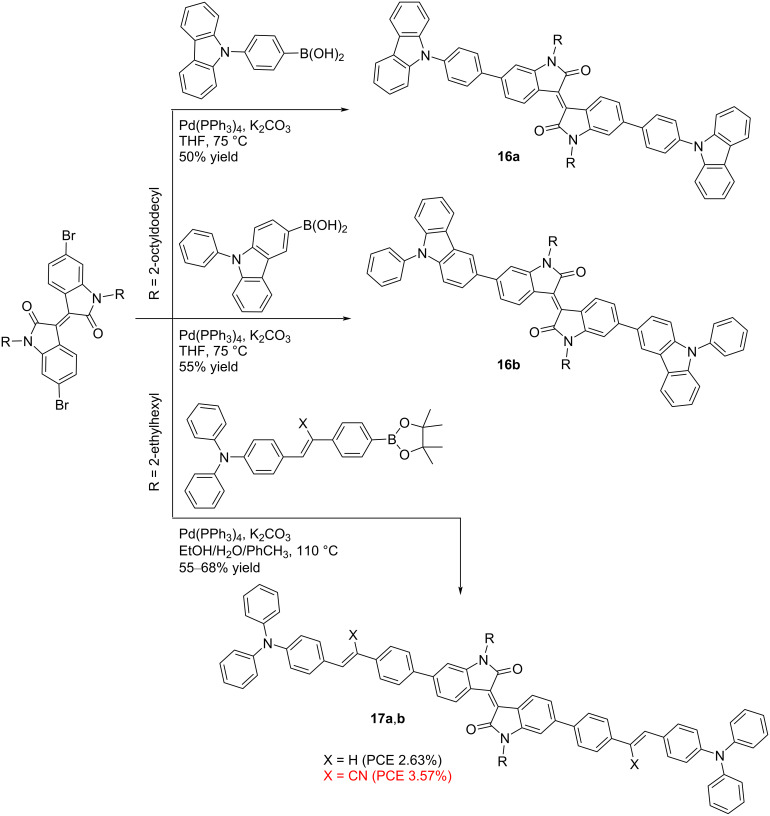
Nonthiophene oligomeric monoisoindigos for OSCs.

It has been shown that oligomeric isoindigos that do not contain a thiophene fragment can also be used as donor components of the OSC. The synthesis of such compounds is also based on the Suzuki coupling reaction between alkylated 6,6’-dibromoisoindigos and corresponding arylboronic derivatives, leading to the formation of target molecules in moderate yield (Scheme 11). In these compounds, the triarylamine substituent is linked either directly to the isoindigo core or via a vinylphenylene bridge (see compounds **17a**,**b**) [27–28]. The presence of the latter determines the cell efficiency to be an order of magnitude higher (3.57%) then when using **16a** and **16b**.

**Scheme 12:**
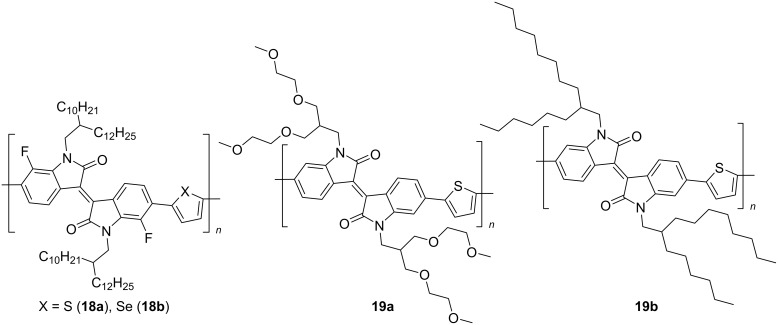
The simplest examples of polymers with a monothienylisoindigo monomeric unit.

In the chemistry of isoindigo-based materials, the most popular and most studied direction in the creation of OSCs is the use of polymer structures containing an isoindigo fragment in a monomer unit associated with a different number of thiophene substituents (Scheme 12).

By the example of one of the simplest representatives of this type of polymers, a significant effect of the structure of the substituent at the nitrogen atom of the heterocycle on the OSC efficiency was also shown [29–30]. Thus, fluorine-substituted polymers **18a**,**b** in the composition with PC_61_BM showed a PCE of only 0.9–1.4%, while an OSC based on ethoxylated derivative **19a** was characterized by a high short-circuit current (*J*_SC_ = 13.92 mA/cm^2^), with a 5-fold better PCE value (Table 3).

**Table 3:** Photovoltaic properties of OSCs based on polymers **18** and **19** with a monothienylisoindigo monomeric unit.

compound	*V*_OC_, V	*J*_SC_, mA/cm^2^	FF, %	PCE, %

**18a**	0.97	2.72	53.1	1.40
**18b**	0.94	1.93	50.0	0.91
**19a**	0.73	13.92	50.2	5.10
**19b**	0.87	4.11	63.4	2.30

**Scheme 13:**
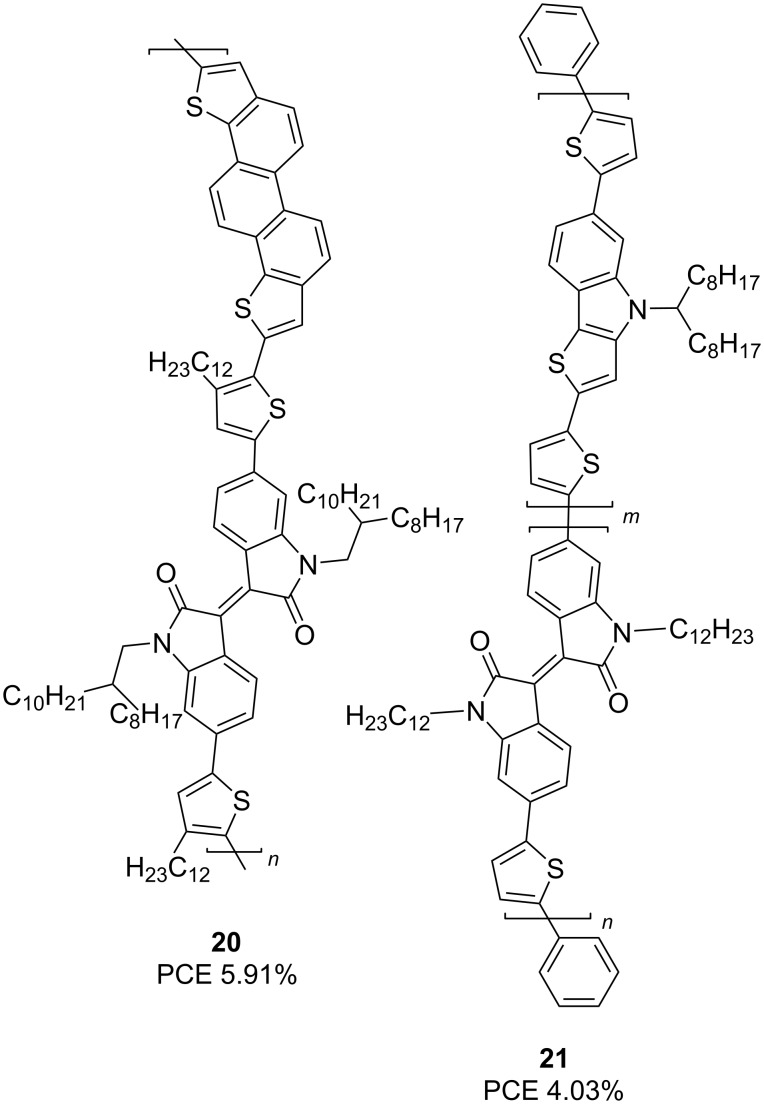
Monothienylisoindigos bearing π-extended electron-donor backbones.

Expansion of the conjugation chain by introducing additional electron-enriched condensed heterocyclic fragments (see compounds **20** and **21**) makes it possible to achieve a PCE of almost 6% (Scheme 13) [31–33].

In the course of further studies, the key influence of the nature of both the substituent at the nitrogen atom of isoindigo and at the bithiophene moiety on the OSC efficiency was confirmed. Thus, the introduction of fluorine atoms to thiophene rings in compound **22b** leads to an increase in the efficiency from 4.58 to 6.21%. Based on the data of quantum chemical calculations, Park et al. [34] showed that in the fluorinated derivative, the dihedral angle between the thiophene rings is 0.88° (for comparison, in compound **22a** it is 17.55°), which provides better planarity of the polymer and, as a consequence, higher velocity transport of electrons under irradiation. Within the framework of this direction, the importance of the method for preparing a thin film of the active layer of OSC was also shown. In reference [35], the OSCs based on a mixture of polymers **23a**,**b** with PC_71_BM and an additive of diphenyl ether (3 vol %) were obtained by spin coating from an *o*-xylene solution. Therein, the fluorinated analog also turned out to be better in terms of the final value of the efficiency. Dithienosylole polymers **24** can also be included in this structure type. Although the authors of reference [36] do not give the exact values of the molecular weight of the obtained polymers, they draw a conclusion about the influence of the molecular weight on the efficiency of the cells (Table 4).

**Table 4:** Molecular weight and photovoltaic properties of OSCs based on polymers **22**–**24**.

compound	R	MW, kDa	*V*_OC_, V	*J*_SC_, mA/cm^2^	FF, %	PCE, %

**22a**	H	45.8	0.87	8.51	61.9	4.58^a^
**22b**	F	75.9	1.01	9.11	67.2	6.21^a^
**23a**	H	79.2	0.89	9.21	60.0	4.92
**23b**	F	91.6	1.06	12.58	66.0	8.80
**24a**	—	not given	0.79	3.34	44.0	1.66
**24b**	—	not given	0.60	1.08	29.0	0.99

^a^After annealing at 200 °C.

**Scheme 14:**
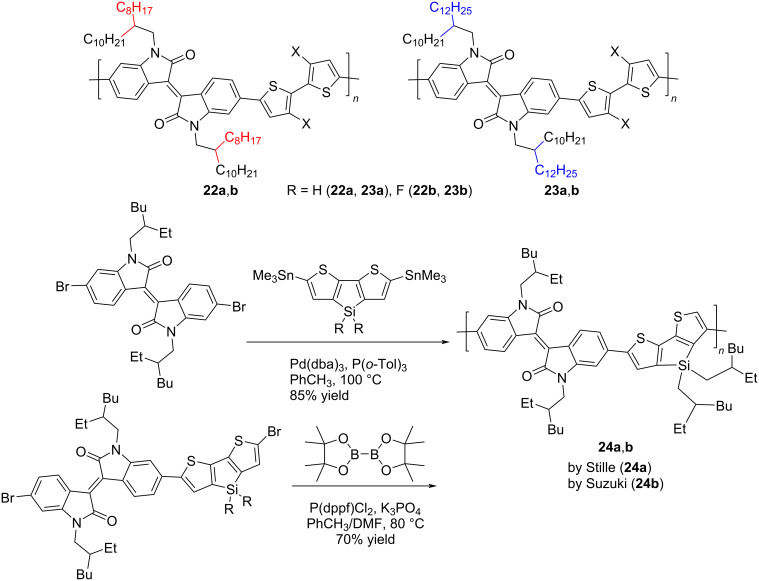
Role of fluorination and the molecular weight on OSC efficiency on the base of the bithiopheneisoindigo series.

Thus, when using polymer **24b** obtained by the Suzuki reaction with bis(pinacolato)diboron, the OSC efficiency was only 0.99%, while the Stille method gave a polymer **24a** characterized by an efficiency of 1.66% (Scheme 14).

**Scheme 15:**
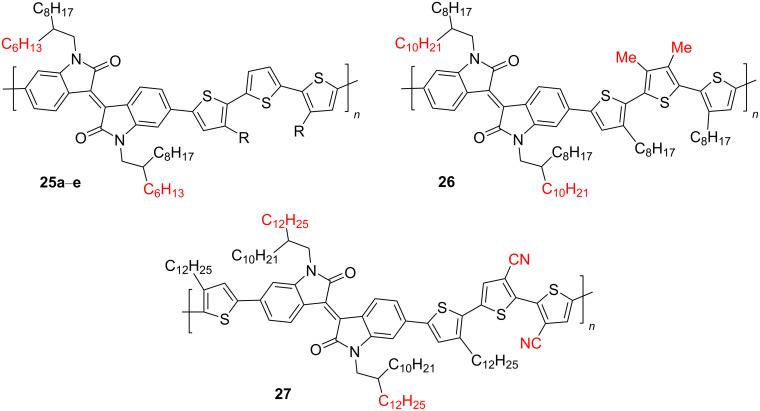
Trithiopheneisoindigo polymers with variation in the substituent structure.

Strengthening the donor effect of the monomer unit can be achieved by lengthening the thiophene chain up to three fragments [37–41]. In this case, additional possibilities arise for fine-tuning the properties of polymers due to the introduction of substituents of different structures in each of the thiophene rings. Using the example of OSCs consisting of a mixture of a polymer **25** and PC_71_BM (1:1.5, w/w), the effect of the length of the alkyl radical on the efficiency of such cells was shown [37]. Thus, the hexyl and octyl derivatives **25a**,**b** showed the best PCE values of 5.1 and 5.2%, respectively, which is higher than analogues bearing a longer or branched hydrocarbon chain (Scheme 15 and Table 5).

**Table 5:** Photovoltaic characteristics of OSCs based on polymers **25**–**27**.

compound	R	*V*_OC_, V	*J*_SC_, mA/cm^2^	FF, %	PCE, %

**25a**	*n*-hexyl	0.69	12.3	58.0	5.1
**25b**	*n*-octyl	0.70	12.7	57.0	5.2
**25c**	*n*-decyl	0.63	11.6	54.0	4.1
**25d**	*n*-dodecyl	0.69	11.3	52.0	4.0
**25e**	2-ethylhexyl	0.88	0.8	58.0	0.3
**26**	—	0.97	5.25	38.0	1.94
**27**	—	1.06	15.7	52.0	8.36

**Scheme 16:**
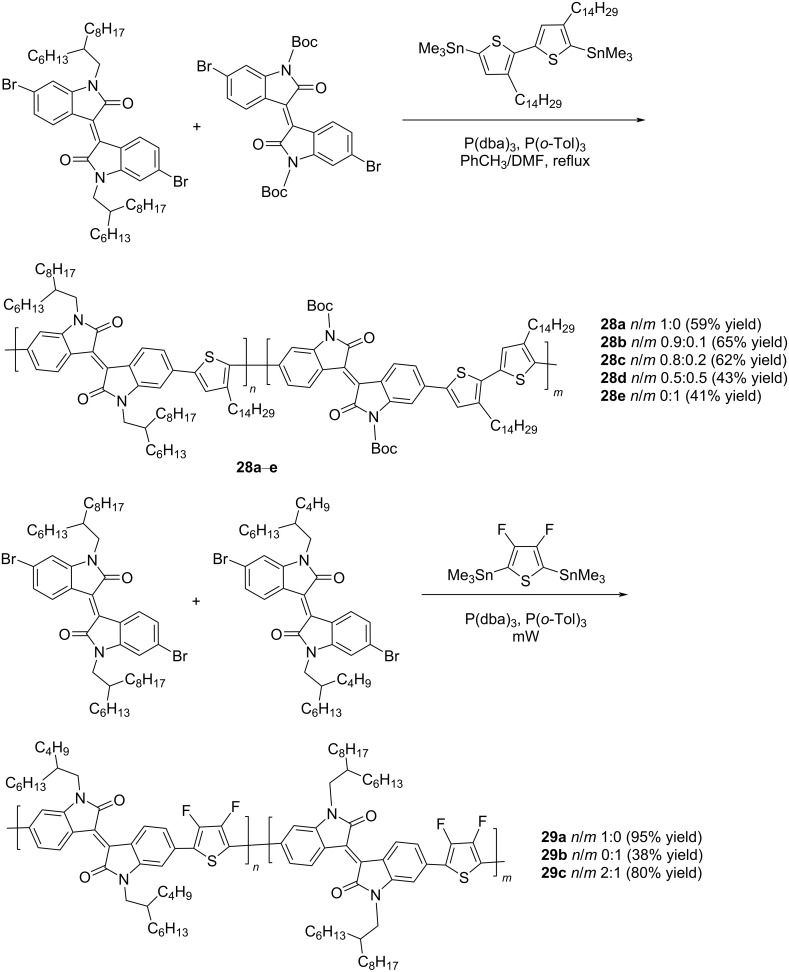
Polymeric thienyl-linked bisisoindigos for OSCs.

If the OSC active layer is prepared with the addition of diiodooctane, the PCE of the octyl analogue **25b** increases to 6.4% [38]. A slight change in the substituent at the nitrogen atom and the introduction of electron-donor methyl or electron-acceptor cyano groups in the thiophene fragment can lead to a sharp deterioration of all characteristics (structure **26**: efficiency 1.94%) [39] or to their significant improvement (structure **27**, efficiency 8.36%), respectively [40]. As it was found, the OSC based on polymer **27** showed one of the best values among the described structures of *V*_OC_ 1.06 V. Others types of polymer structures that have good potential in organic photovoltaics can be found in the form of derivatives **28** and **29**, containing two differently substituted isoindigo fragments connected through a thienylene spacer (Scheme 16).

Bini et al. described the synthesis of polymer series **28a**–**e** with different ratios of isoindigo fragments, one of which is capable of thermal cleavage of Boc groups [42]. Without studying the main characteristics of the OSCs, they showed that after annealing the mixtures of these polymers with PC_61_BM at 200 °C, the surface morphology becomes highly inhomogeneous, with the presence of a large number of crystalline domains. In contrast to the above described study, Liu et al. demonstrated that this type of compounds is promising by means of introducing fluorine atoms into the thiophene ring [43]. The use of compound **29c** (ratio of monomer units *n*/*m* = 2:1) as an acceptor component of the OSC made it possible to achieve one of the highest efficiency values of 7.3%. The problem of the low solubility of such polymers was partially solved by inserting an alkylene spacer between two thiophene fragments in one of the monomer units [44]. Efficiency (3.0–3.7%) and viscosity characteristics provide good prerequisites for the use of this type of polymers in the design of flexible OSCs.

**Scheme 17:**
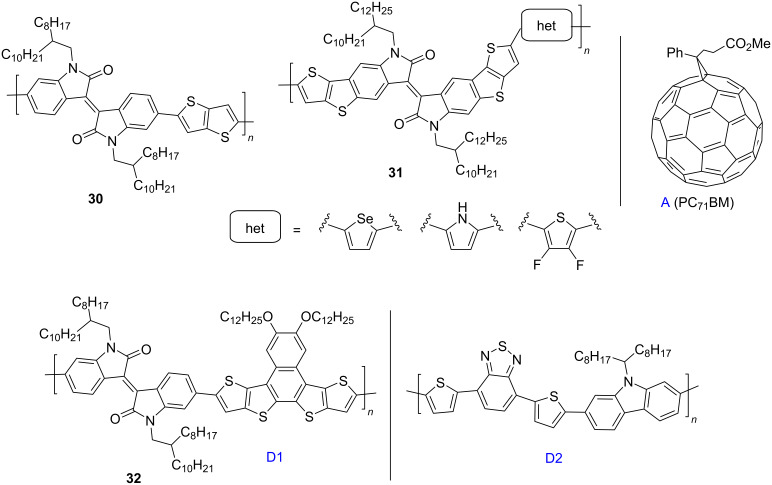
Isoindigo bearing the thieno[3,2-*b*]thiophene structural motif as donor component of OSCs.

Condensed thienothiophene substituents can also be used as the donor component of the monomeric isoindigo unit (Scheme 17). The first data on the use of these compounds as donor components of OSCs (mixed with PC_61_BM) showed that the technology of preparing a thin film of the active layer is important to achieve the best efficiency value [45]. Thus, the best results using compound **30** (efficiency 2.24%) were shown by a cell with an active layer of 44 nm obtained by shifting the solution along the substrate at a rate of 0.1 mm/s. Compared to compound **30**, OSCs based on more complex condensed analogs **31** containing a heterocyclic fragment showed a 2-times better efficiency (5.6% for difluorothiophene, 5.0% for selenophene) [46].

The effect of the length and branching of the alkyl substituent at the endocyclic nitrogen atom in a series of this type of donor polymers was investigated. The 2-hexyldecyl derivative exhibited the best compatibility with PC_71_BM, which resulted in a high PCE value (6.83%) of the corresponding OSC [47]. This efficiency may be due to the good surface morphology of the composite thin film and, as a consequence, the high short-circuit current (*J*_SC_ = 13.55 mA/cm^2^). It is interesting to note the direction in which isoindigo polymers are used in the design of ternary systems of the OSC active layer. Thus, an OSC consisting of a composite based on two donor polymers (D1 and D2) and an acceptor component PC_71_BM (A) with a D/A weight ratio of 1:4 showed an efficiency of 7.69% with a D1 content of 15% relative to the D2 weight. It should be especially noted that the efficiency of the cell without isoindigo D1 was 6.91%.

**Scheme 18:**
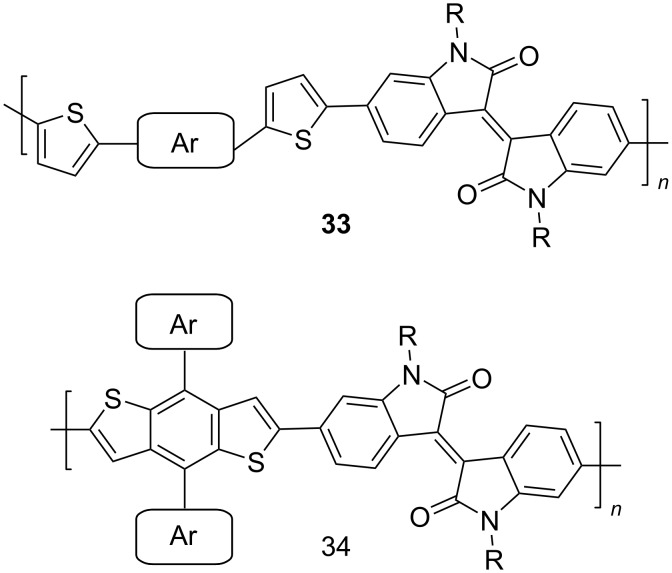
Thienylisoindigos with incorporated aromatic unit.

To increase the degree of conjugation in the structure of polymeric isoindigo, an introduction of additional aromatic fragments either into the main monomeric chain (compounds of type **33**) [48–50] or as a side substituent in a thiophene unit (compounds of type **34**) [51–53] was proposed. Among a number of compounds **33**, the best efficiency (5.29%) was shown by an OSC based on a mixture of PC_71_BM and polymer **33с** containing a short *n*-butyl substituent at the isoindigo nitrogen atoms and the longest and most branched alkyl radical in the *p*-phenylene fragment (Scheme 18).

Sun et al. showed that such a combination of substituents provides the best miscibility of the polymer with the acceptor fullerene component, which accordingly improves the morphological and photophysical characteristics of the OSC [50]. For comparison, it should be noted that the OSC based on phenanthroquinoxaline derivative **33d** showed an efficiency of only 0.3%. The study of composites based on mixtures of compounds **34** with PC_71_BM demonstrated the advantages of the presence of an alkoxyphenyl substituent in the side chain, providing an efficiency of 5.23%. Therein, the presence of a substituent in the phenylene fragment is also an important factor (Table 6). Thus, an OSC based on a fluorine-containing polymer showed an efficiency of only 0.93%. This may be due to the low hole conductivity, a decrease in the HOMO level of the polymer, and a narrower band gap of visible light absorption [51].

**Table 6:** Photovoltaic characteristics of OSCs based on aromatic embedded polymers **33** and **34**.

compound	polymer	*V*_OC_, V	*J*_SC_, mA/cm^2^	FF, %	PCE, %

**33a**	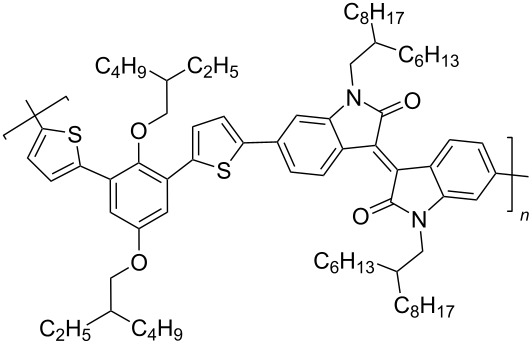	0.71	2.7	35.0	0.67
**33b**	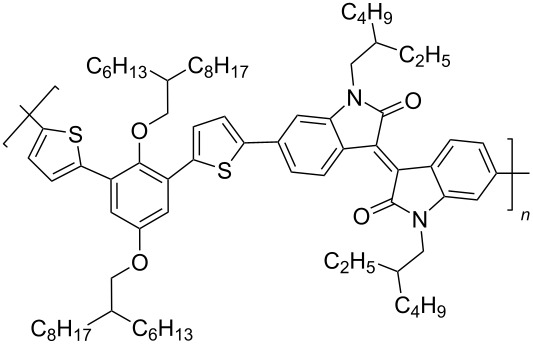	0.87	10.1	43.0	3.79
**33c**	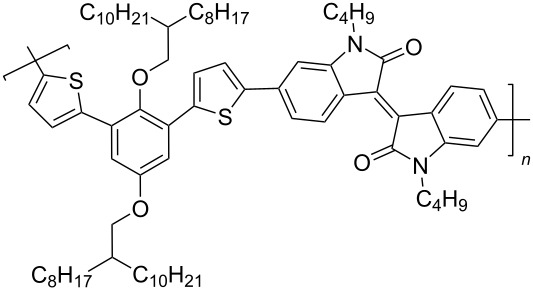	0.81	11.7	56.0	5.29
**33d**	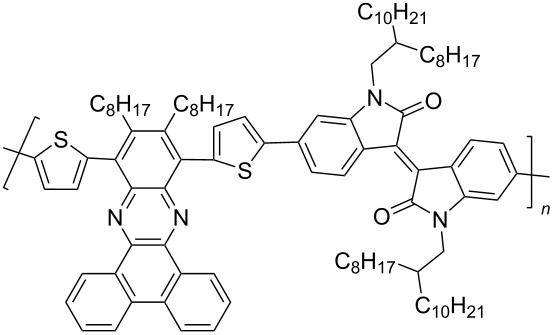	0.86	1.2	30.0	0.3
**34a**	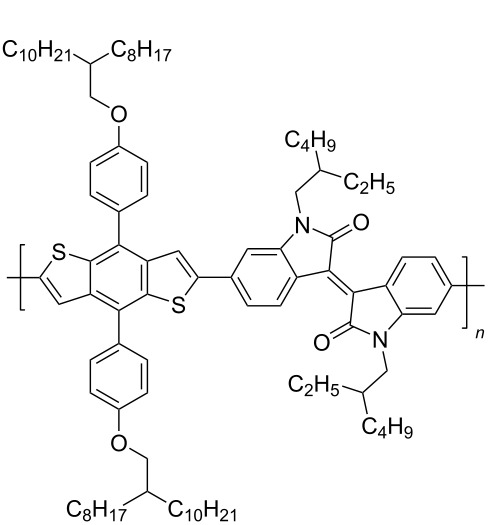	0.84	9.7	64.0	5.23
**34b**	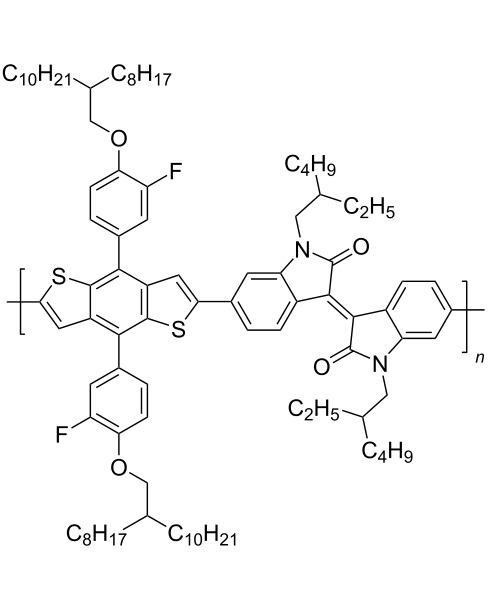	0.88	5.6	50.0	2.50
**34c**	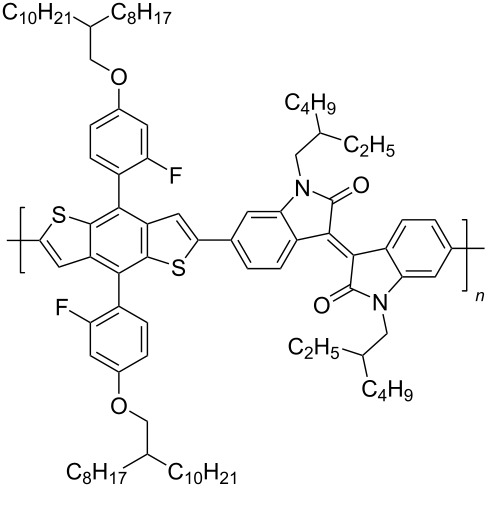	0.96	1.6	60.0	0.93
**34d**	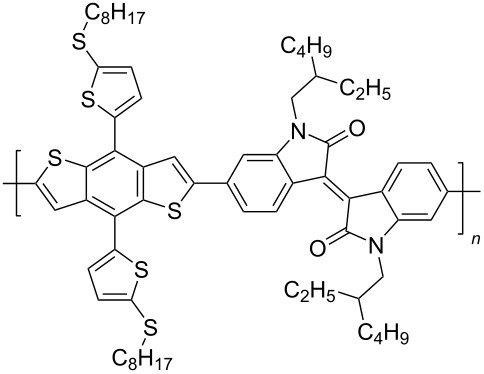	0.84	8.2	39.0	2.70

**Scheme 19:**
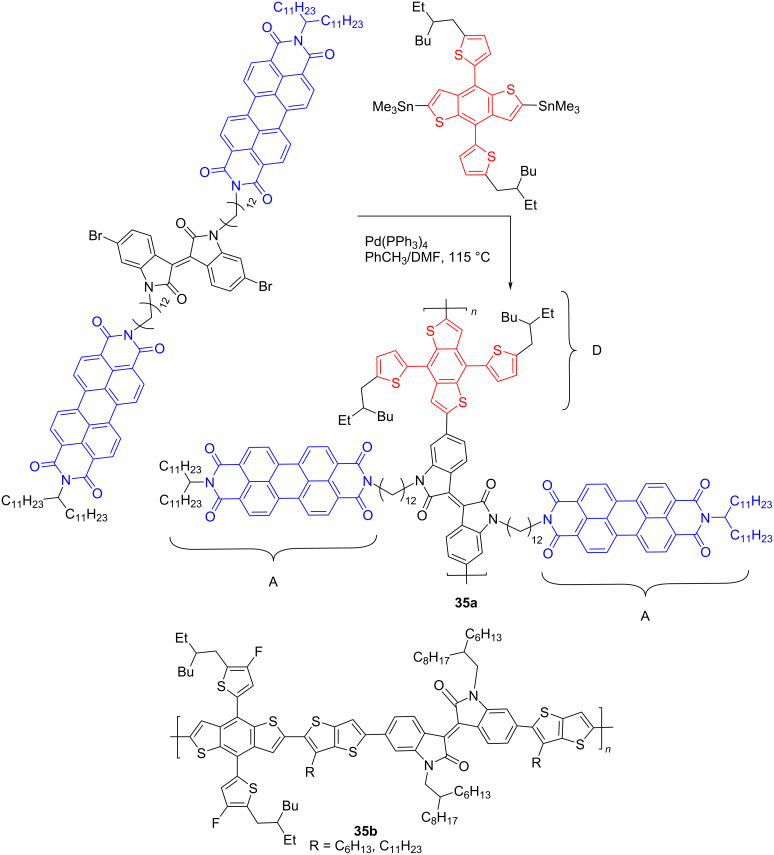
One-component nonfullerene OSCs on the base of isoindigo.

Within the framework of the study of the prospects for polymer derivatives of isoindigo in organic photovoltaics and avoiding the use of the fullerene components, the concept of creating one-component OSCs appeared [54]. Following this strategy, isoindigo was used as platform for the synthesis of compound **35a**, combining acceptor (perylene diimide) and donor (polythiophene) fragments in the structure. Both polymers were obtained using the Stille cross-coupling reaction (Scheme 19). However, the OSC of such a cell showed an efficiency of only 1%. Using polymeric isoindigo **35b** as an acceptor component, a nonfullerene OSC was also obtained, which showed a record efficiency of 12.03% among the composites based on isoindigo described to date [55].

**Scheme 20:**
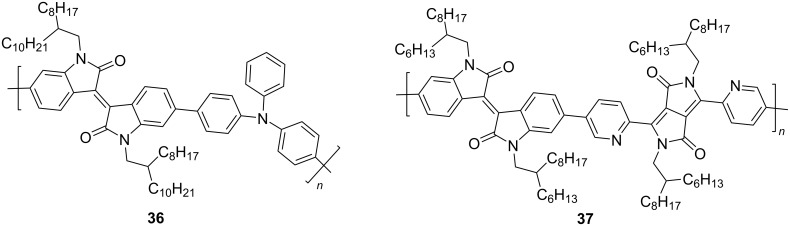
Isoindigo-based nonthiophene aza aromatic polymers as acceptor components of OSCs.

Polymeric derivatives of isoindigo containing no thiophene units were also used as acceptor components of the OSCs. Moreover, in both of the studies described in recent years, the donor component of the photovoltaic cell was variously substituted polythiophene, while the acceptor component (isoindigo platform) was functionalized with aromatic nitrogen-containing substituents of various structures (Scheme 20). The design of DPP copolymers of isoindigo **37** seems to be the most promising here [56] since OSCs based on it showed a higher efficiency than with compound **36** (4.2 vs 0.26%) [57].

**Scheme 21:**
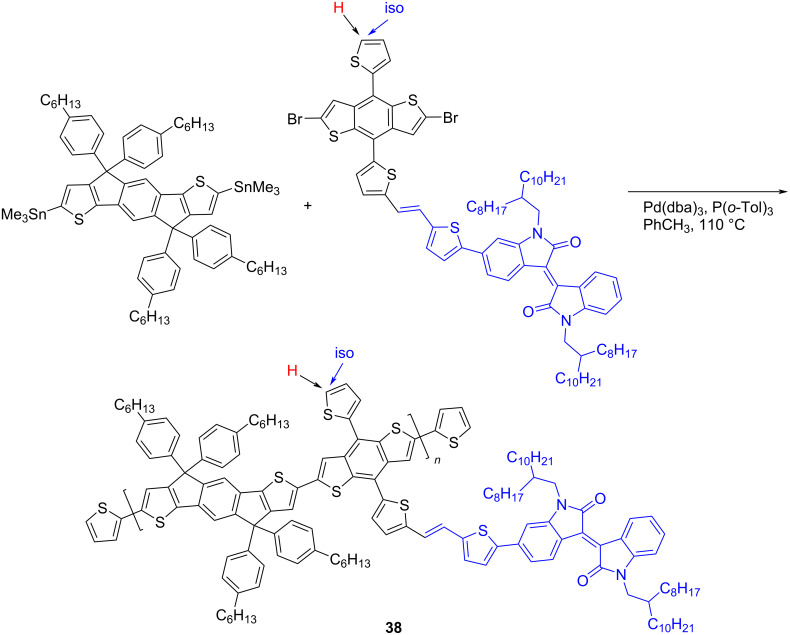
Polymers with isoindigo substituent as side-chain photon trap.

Considering the indigoid bisheterocycle as an additional photon trap, the substituted isoindigo was introduced as an acceptor substituent in the structure of the polyconjugated thiophene polymer **38** [58]. For this purpose, two indacenothiophene polymers were obtained containing one or two isoindigo fragments (the position of the introduction of the second isoindigo fragment is indicated by an arrow, Scheme 21). However, the efficiency of the cell based on the monoindigo derivative turned out to be slightly higher (2.66 vs 2.50%).

### Isoindigo as the basis for organic field-effect transistors (OFET)

**Scheme 22:**
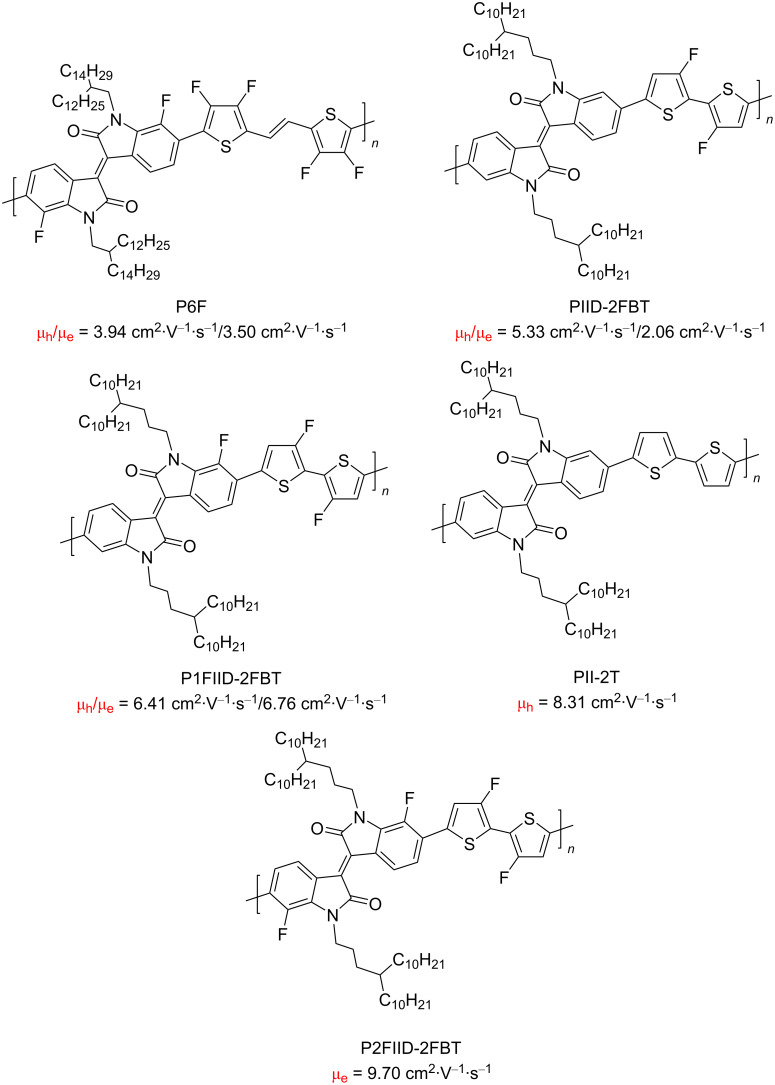
Isoindigo derivatives for OFET technology with the best mobility.

In recent years, the interest of researchers in semiconductor materials has covered the field of organic electronics. The advantages of organic oligomeric and polymeric materials for applications in OFET and thin-film transistor (TFT) technologies are due to the ease of their directed chemical modification, mechanical flexibility, the possibility of varying optoelectronic properties, and good solubility in a wide range of solvents [59–61]. In this regard, the most studied and promising are π-conjugated polymer structures based on sulfur, oxygen, nitrogen, and selenium heterocyclic compounds [62–63]. One of the systems for creating OFET devices is the isoindigo platform [64–68]. Scheme 22 shows polymeric structures with the best mobility values among isoindigo derivatives that are known to date.

**Scheme 23:**
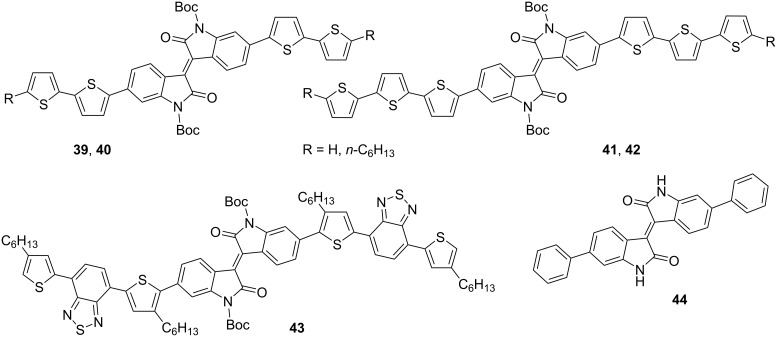
Monoisoindigos as low-molecular-weight semiconductors.

Compounds **39**–**44** are the simplest representatives of isoindigo derivatives used in the design of OFET devices (Scheme 23). In a series of compounds of this type, the central platform of isoindigo is substituted in positions 6,6' either by a phenyl or by a substituted thiophene fragment. Thus, Ashizawa et al. [69] established the ambipolar character of the conductivity of 6,6'-diphenylisoindigo with μ_h_/μ_e_ = 0.037 cm^2^·V^−1^·s^−1^/0.027 cm^2^·V^−1^·s^−1^. Although the obtained values of mobility turned out to be low, this finding could have contributed to the development of this direction by the design of electron-donating aromatic substituents at the isoindigo core. However, as was shown in subsequent works, neither a change in the length and structure of the thiophene chain, nor the presence of an embedded benzothiadiazole fragment led to an improvement in the characteristics of transistors based on compounds **39**–**43** [70–73]. These derivatives had only hole-type conductivity in the range μ_h_ = 1.5⋅10^−4^–6⋅10^−6^ cm^2^·V^−1^·s^−1^.

**Scheme 24:**
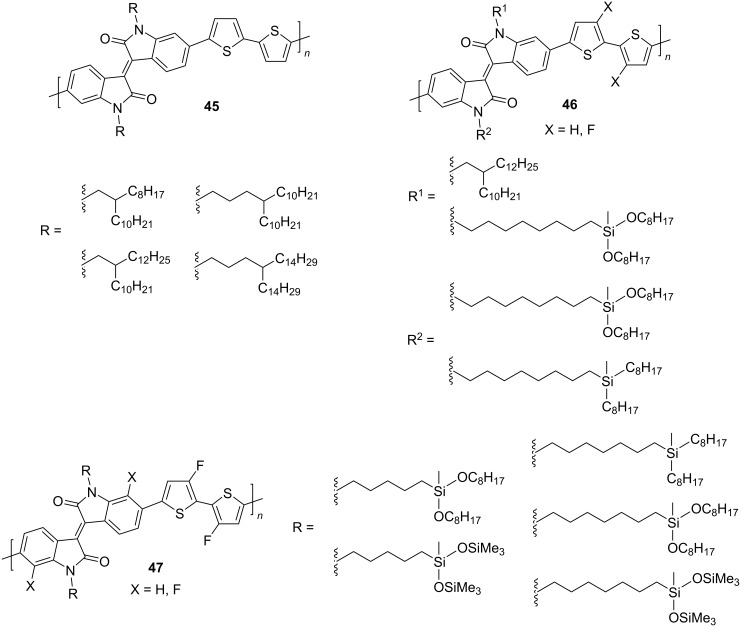
Polymeric bithiopheneisoindigos for OFET creation.

According to the available data, the most studied polymeric isoindigos are dithienyl derivatives, in which two thiophene fragments can be linked to each other [74–86] or be separated by spacers of different structures [87–91]. Structures containing a 2,2'-dithienyl fragment can be divided into three types: 1) not containing substituents in the benzo and thienyl fragments; 2) containing fluorine atoms in the thienyl fragment; and 3) containing fluorine atoms in positions 7,7' and in thienyl fragments (Scheme 24).

Studies of the characteristics of OFETs based on compounds **45** showed almost complete absence of a dependence of the semiconductor properties (μ_e_ = 0.08–0.01 cm^2^⋅V^−1^⋅s^−1^) on the structure of the alkyl substituent at the nitrogen atom. It was only established that the distancing of the branching position of the alkyl chain affects the ordering of molecules in a thin polymer film after annealing [83]. At the same time, for the example of polymer **45** containing a 4-decyltetradecyl radical, it was shown that the efficiency of an OFET device depends on the method of thin film processing. In contrast to the traditional spin-coating technique, the authors succeeded in obtaining a thin film by immersing a substrate in a polymer solution, followed by slow extraction, accompanied by slow evaporation of the solvent. The device thus obtained showed one of the highest values of hole conductivity of μ_e_ = 8.3 cm^2^⋅V^−1^⋅s^−1^ [78]. It was also found that a device based on compound **45** (R = 2-decyltetradecyl) with the addition of iron phthalocyanine showed slightly better mobility. Liu et al. explain this effect by an improvement in the hole-type conductivity and a tight and even packing of the composite in a thin film [80]. At the same time, selenophene analogs generally show lower values of mobility [90]. The introduction of fluorine atoms into the dithienyl fragment (see compound **46**), while varying the symmetry of substitution of the 1,1' positions in isoindigo, did not lead to an improvement in the OFET efficiency (maximum μ_h_ = 1.08 cm^2^⋅V^−1^⋅s^−1^). For comparison, a similar device based on a nonfluorinated analog showed a value of μ_h_ = 2.71 cm^2^⋅V^−1^⋅s^−1^ [74]. Continuing the study of the effect of the substituent nature on the OFET efficiency, a number of polymers **47** with varying degree of fluorination of the monomer structural units was obtained [79]. The study showed that the transition from a less fluorinated analogue (two fluorine atoms on dithiophene) to one containing a larger number of fluorine atoms (two fluorine atoms on dithiophene and two in the 7,7' positions) improves the planarity of the structural units of the polymer and increases the degree of crystallinity, which consequently increases μ_e_. At the same time, the polymer containing two fluorine atoms on the dithiophene unit and one in position 7 exhibited balanced ambipolar properties, having the currently best ratio of μ_h_/μ_e_ = 6.41 cm^2^⋅V^−1^⋅s^−1^/6.46 cm^2^⋅V^−1^⋅s^−1^ among isoindigoid polymers.

**Scheme 25:**
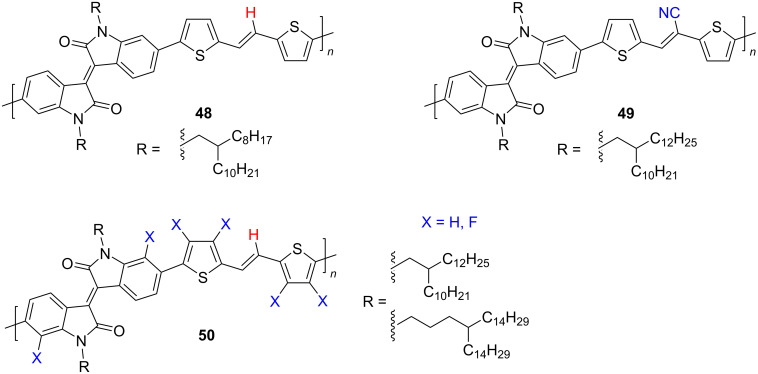
Fluorination as a tool to improve isoindigo-based OFET devices.

Attempts to improve the efficiency of isoindigo-based OFETs by introducing an ethylene bridge between thiophene fragments in general did not lead to the desired result (Scheme 25).

Thus, devices based on polymers **48** and **49** showed an analogy in the unipolar character of the conductivity with close values of μ_h_ = 0.68–0.83 cm^2^⋅V^−1^⋅s^−1^ [87,91]. It should be noted here that more detailed studies on selenium analogs of polymers **48** are promising since the latter showed moderate values of electronic mobility of μ_e_ = 1.28 cm^2^⋅V^−1^⋅s^−1^ [89]. It was shown that a significant improvement of the semiconducting properties of an isoindigo polymer can be achieved by “multifluorination”, the introduction of fluorine atoms both to the dithiophene fragment and to the 7,7'-positions of isoindigo (compound **50**) [88]. As such, the presence of fluorine atoms led to the ambipolarity of the polymer with an effective ratio μ_h_/μ_e_ = 3.94 cm^2^⋅V^−1^⋅s^−1^/3.50 cm^2^⋅V^−1^⋅s^−1^.

**Scheme 26:**
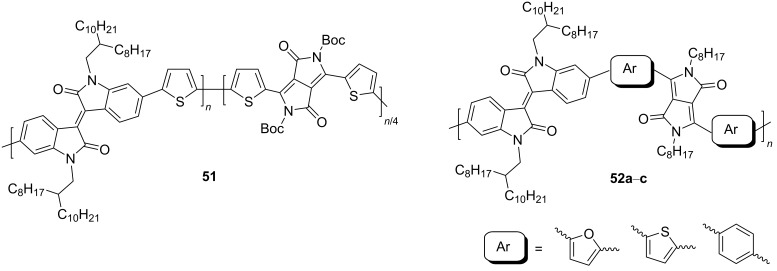
Diversely DPP–isoindigo-conjugated polymers for OFETs.

Recently, diketopyrrolo[3,4-*c*]pyrrole (DPP) derivatives, which are highly conjugated electron-withdrawing heterocycles with high charge conductivity, broad absorption spectrum, photostability, and thermal stability have attracted considerable interest of researchers in the field of organic electronics [92–94]. Isoindigo derivatives have similar characteristics. Taking into account these data, copolymers **51** containing up to 25% DPP units were obtained [95]. Despite the good prerequisites, an OFET based on this copolymer showed only hole-type conductivity with μ_h_ = 1.2⋅10^−3^ cm^2^⋅V^−1^⋅s^−1^. Therein, the thermolysis of a thin film of the device at 220 °C, accompanied by the elimination of Boc groups, led to a significant decrease of the OFET performance. For the example of the polymers **52** series, the importance of the spatial arrangement of the isoindigo and DPP fragments relative to each other was demonstrated [96]. Thus, the dihedral angle 179° in a furan polymer determines the conductivity μ_h_/μ_e_ = 0.01 cm^2^⋅V^−1^⋅s^−1^/1.6⋅10^−3^ cm^2^⋅V^−1^⋅s^−1^, while polymers containing thiophene or *p*-phenylene spacers did not possess conductivity at all due to the lower planarity (dihedral angle 143°, Scheme 26, Table 7).

**Table 7:** Transistor characteristics of DPP–isoindigo-conjugated polymers **51** and **52**.

compound	polymer	μ, cm^2^⋅V^−1^⋅s^−1^	

		hole	electron

**51**	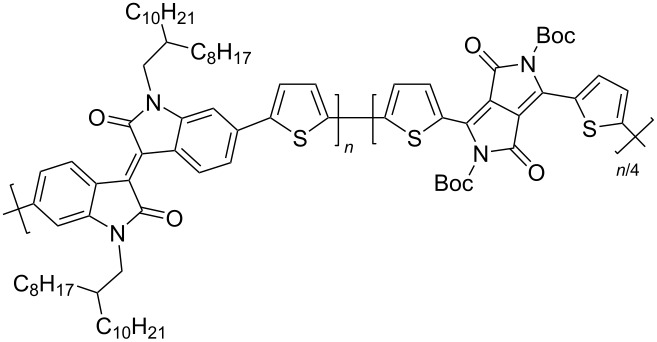	6.7⋅10^−8^	—
**52a**	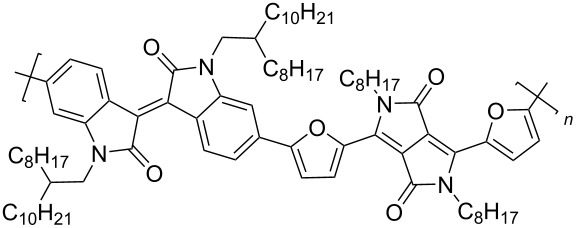	0.01	1.6⋅10^−3^
**52b**	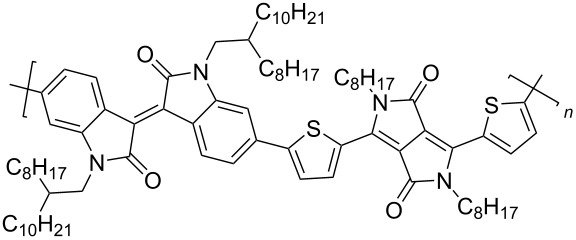	8.4⋅10^−3^	2.0⋅10^−3^
**52c**	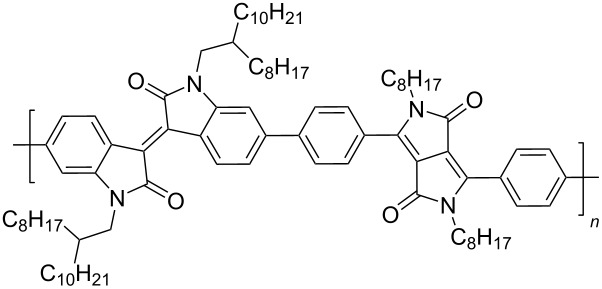	—	—

**Scheme 27:**
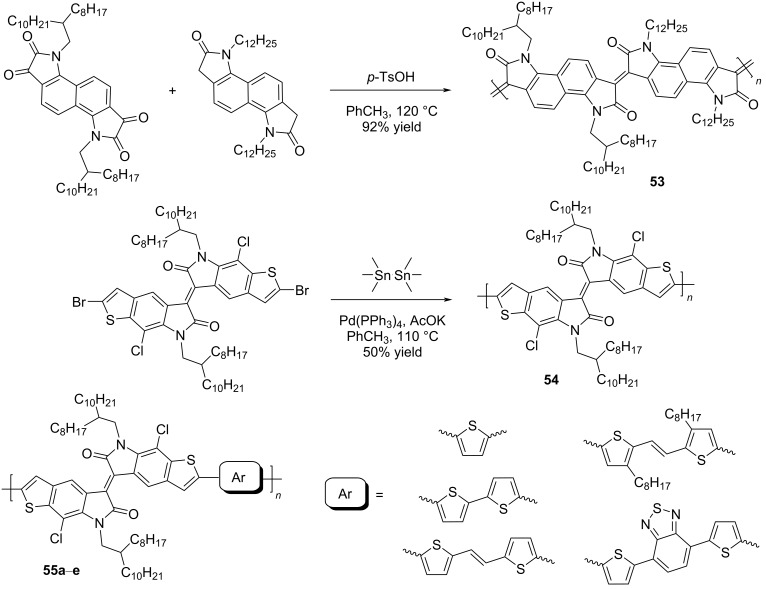
Isoindigoid homopolymers with differing rigidity.

Homopolymers based on isoindigo of various types have also been used in the design of transistors [97–100]. In order to reduce the influence of factors of conformational and energy disordering inherent in all isoindigo polymers in which aromatic fragments are linked by a single bond, a number of homopolymers **53** was obtained by the aldol polycondensation reaction [97]. These compounds have rigid and almost planar structures with a wide absorption range, high electron affinity, good solubility, and ambient stability (Scheme 27). The study of the transistor characteristics showed that these homopolymers have an electronic type of conductivity, with a maximum value of μ_e_ = 0.03 cm^2^⋅V^−1^⋅s^−1^. The work [98] was also aimed at obtaining OFET devices based on homopolymeric isoindigo. The thieno-based condensed polymers **54** described herein, in which the monomeric isoindigo fragments are linked by a single bond, were obtained in two ways: by Suzuki (40% yield) and Stille (50% yield) coupling reactions. Despite the possibility of rotation of monomeric fragments around a single bond, a transistor based on this polymer showed relatively high values of mobility of μ_h_/μ_e_ = 0.065 cm^2^⋅V^−1^⋅s^−1^/0.15 cm^2^⋅V^−1^⋅s^−1^. It is also worth presenting data on compounds **55** containing thiophene bridges of various structures between the isoindigo nuclei [99,101]. Therein, the presence of a spacer, in comparison to compounds **54**, led to a decrease of the OFET performance (maximum μ_h_/μ_e_ = 0.037 cm^2^⋅V^−1^⋅s^−1^/0.029 cm^2^⋅V^−1^⋅s^−1^, Table 8).

**Table 8:** Transistor characteristics of homopolymers **53**–**55**.^a^

compound	polymer	μ, cm^2^⋅V^−1^⋅s^−1^	

		hole	electron

**53**	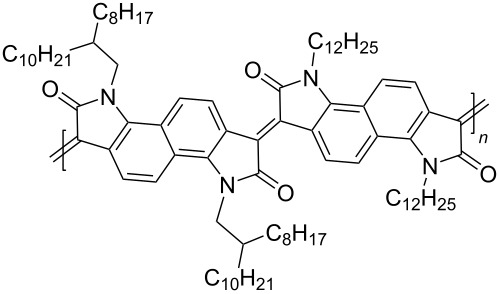	—	0.03
**54**	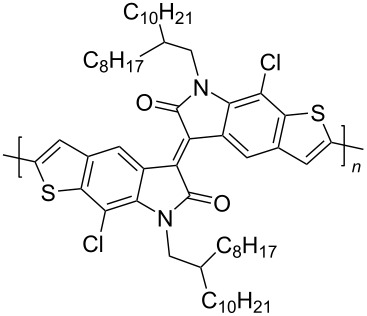	0.065	0.15
**55a**	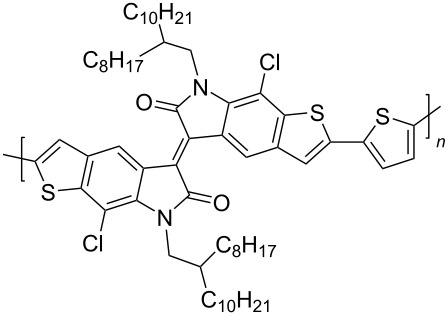	0.018	0.029
**55b**	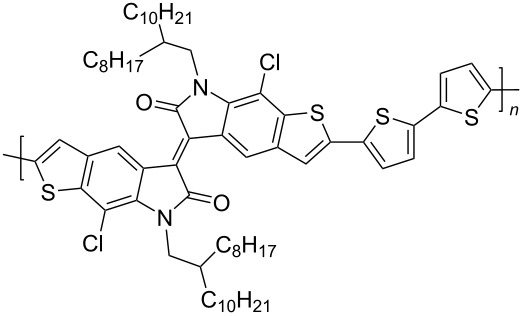	—	—
**55c**	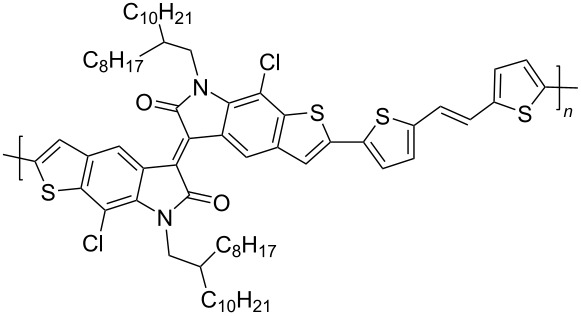	0.037	0.004
**55d**	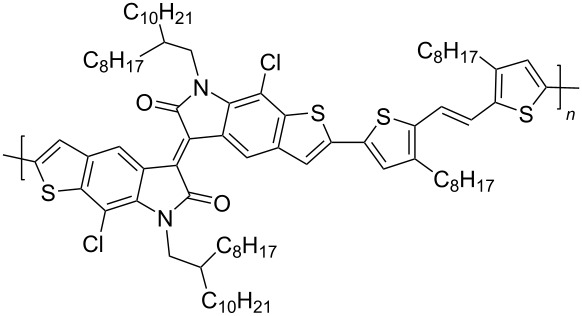	—	—
**55e**	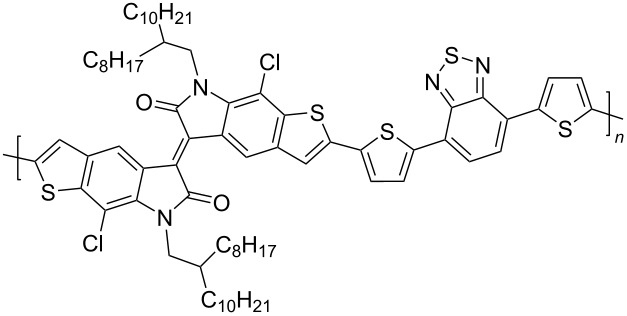	—	—

^a^Polymers **55b**,**d**,**e** were not studied due to the low solubility in a wide range of organic solvents.

**Scheme 28:**
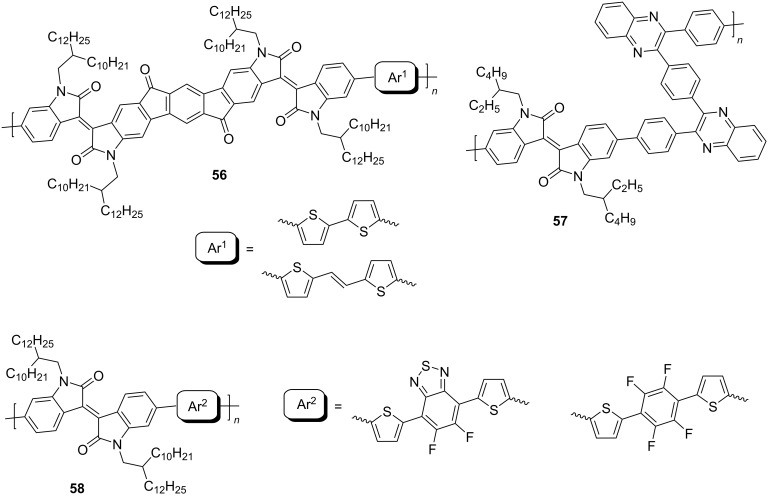
Isoindigo-based materials with extended π-conjugation.

One of the directions for the design of polymeric isoindigo derivatives to improve the conductive properties is the lengthening of the conjugation chain of both the monomer unit itself and the monomer subunit [102–105]. Thus, two polymers **56** were obtained in which isoindigo fragments are condensed on the indacenedione scaffold [102]. Despite the presence of an extended π-conjugation system, which determines the ambipolar properties of a transistor based on these polymers, the charge mobility values turned out to be rather low (Scheme 28). The maximum value of μ_h_/μ_e_ = 0.1 cm^2^⋅V^−1^⋅s^−1^/0.14 cm^2^⋅V^−1^⋅s^−^1 was shown by a polymer containing a bridging ethylene fragment between two thiophene substituents. The lengthening of the conjugation chain within the monomeric unit can be achieved by introducing phenylenequinoxaline [103] or fluorinated phenylenethiophene [104] fragments. Based on compound **57**, a flexible OFET with μ_e_ = 0.25 cm^2^⋅V^−1^⋅s^−1^ was fabricated on a 3D printer. In contrast to the above, an OFET based on difluorobenzothiadiazole polymer **58** showed hole conductivity with a low charge mobility value of μ_h_ = 0.07 cm^2^⋅V^−1^⋅s^−1^.

### Isoindigo-based sensor devices

**Scheme 29:**
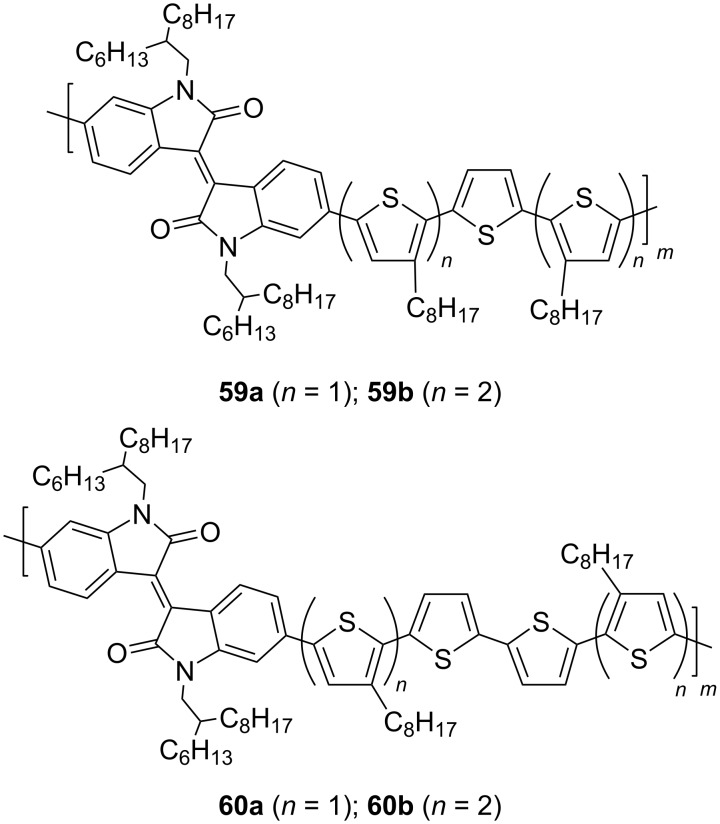
Poly(isoindigothiophene) compounds as sensors for ammonia.

Isoindigo derivatives have begun to find an application in the design of sensor devices for the detection of simple and complex molecules in various aggregation states. Thus, it was shown that polymer structures based on isoindigo and thiophene **59a**,**b** and **60a**,**b** are able to effectively bind gaseous ammonia molecules (Scheme 29) [106].

Thin films of these polymers are characterized by high sensitivity, reproducibility, and fast response time. Among this series of compounds, polymer **60b**, containing a terthiophene oligomeric unit, has the best characteristics. Further studies showed that a polymer composite based on the terthiophene analogue **59a**, poly(methyl methacrylate), and polyaniline is capable of detecting vapors of some organic solvents (chlorobenzene, *n*-butanol, DMF, isopropanol, and toluene) [107]. Therein, the best sensitivity was found towards *n*-butanol. The lower limit of detection was 100 ppm with a response time of less than 10 s.

**Scheme 30:**
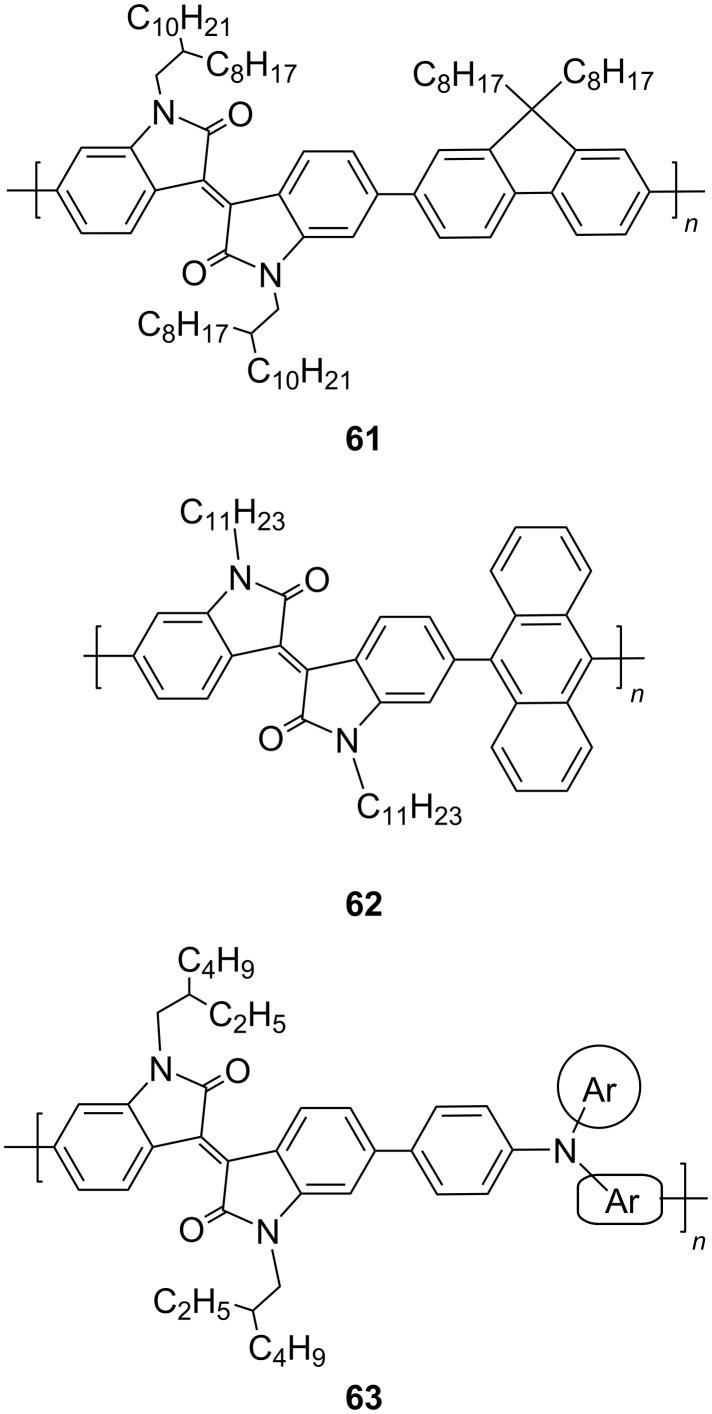
Sensor devices based on poly(isoindigoaryl) compounds.

The ability of polymeric isoindigo derivatives to strongly bind to carbon nanotubes due to π-stacking was used to create sensors for the determination of NO_2_ in the gaseous state (see polymer **61**) and glucose in solution (see polymer **62**) [108–109]. The limit of sensitivity of the sensor for nitrogen dioxide was 60 ppm and for glucose 0.026 mM. For the example of the fairly simple arylamine series **63**, the possibility of using isoindigo polymer for the detection of explosives (trinitrophenol and trinitrotoluene) in solution was demonstrated (Scheme 30) [110–111].

### Miscellaneous applications

Taking into account the high thermal, atmospheric, mechanical, and redox stability of isoindigo polymers, various scientific groups focused their studies on the development of new directions for the practical application of these materials. Thus, Gu et al., using the example of a donor–acceptor–donor (D–A–D) polymer **64** containing a 3,4-ethylenedioxythiophene fragment, demonstrated the possibility of creating flexible IR displays based on isoindigo [112]. This polymer showed very good electrochromic characteristics, such as staining efficiency (362 cm^2^⋅C^−1^ at 1050 nm), fast switching time (0.5 s), high optical contrast (59% at 1500 nm), and redox stability (<8% after 4000 cycles).

Poly(isoindigothiophene) **65** containing sulfonate groups in the side chain was used as an anionic photoactive polyelectrolyte in a composite with platinum nanoparticles stabilized with poly(acrylic acid) [113]. Such a catalytic system, obtained by layer-by-layer self-assembly with the addition of poly(diallyldimethylammonium chloride) on the indium tin oxide surface, provided hydrogen formation in photoelectrolytic cycles with a Faraday yield of about 45%.

The search for new stable polyfunctional materials for the mass spectrometric determination of low-molecular-weight compounds led the authors of reference [114] to the discovery of new properties of polyisoindigo **66**. It was found that this polymer can be used as a two-mode matrix (in positive and negative modes), which is a rarity for the MALDI method. The detection limits were below 164 pmol for reserpine and below 245 pmol for cholic acid.

**Scheme 31:**
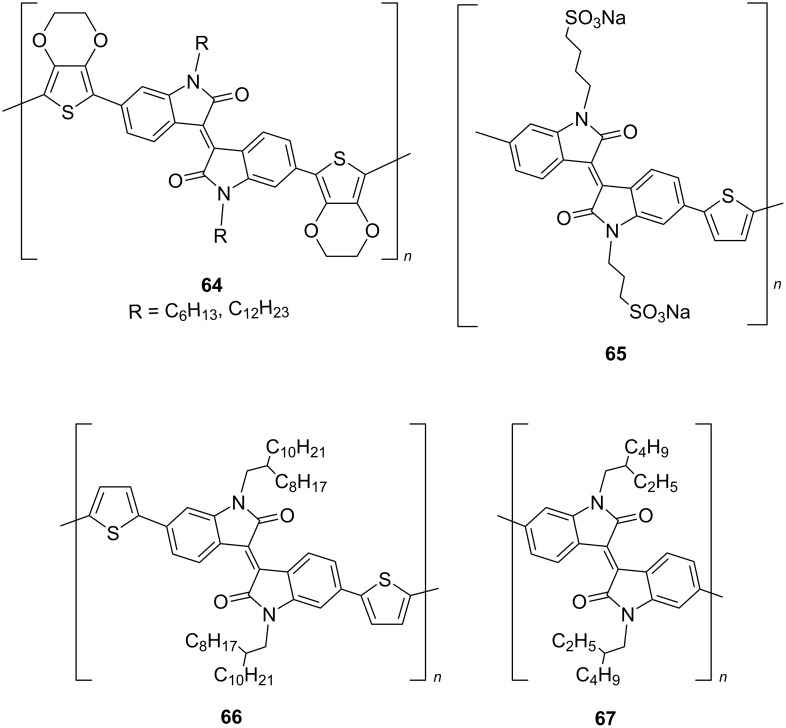
Isoindigo polymers for miscellaneous applications.

More recently, another new application of polyisoindigos was discovered as a new conductive binder inside electrodes containing silicon nanoparticles coated with a carbon shell (Si@C) for lithium ion batteries [115]. The specific capacity of a battery designed using polyisoindigo **67** (up to 1400 mA⋅h/g) with high stability (up to 500 cycles) indicates a high potential of such structures in the search for alternatives to the existing polymer conductive binder mixed with carbon additives (Scheme 31).

**Scheme 32:**
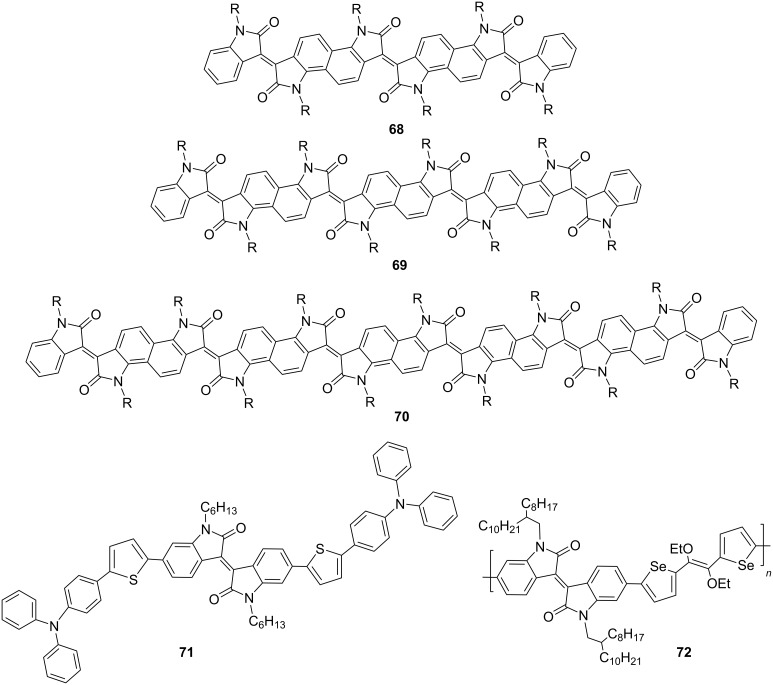
Mono-, rod-like, and polymeric isoindigos as agents for photoacoustic and photothermal cancer therapy.

Isoindigo derivatives have begun to find use in biomedical applications. Thus, nanoparticles of isoindigoid polymers have shown good potential as agents for photoacoustic and photothermal cancer therapy [116–121]. In this field, a number of condensed derivatives of oligoisoindigo **68**–**70**, triphenylamine-containing monoisoindigo **71**, and selenophenevinylene polymer **72** were investigated (Scheme 32). In terms of photothermal conversion (62–71% yield), ribbon-like compounds turned out to be the most effective [120]. At the same time, in vivo experiments have shown the high efficiency of low-molecular-weight isoindigo **71** in oxygen sensitization for cancer therapy [121]. Therein, a high value (84%) of the singlet oxygen quantum yield was obtained.

## Conclusion

To summarize, it can be concluded that isoindigo is a promising platform for creating materials for various purposes – from organic solar cells and transistors to materials for biomedical applications. The possibility of easy modification and easy accessibility of the starting reagents for the synthesis of polysubstituted isoindigo derivatives provides the possibility of fine-tuning the properties and wide design of this bisheterocycle. In particular, to improve the characteristics of organic solar cells and OFET devices based on polymer derivatives of isoindigo, some of the most important factors are the planarity of the monomer unit, the electron donor/acceptor nature, the heterocyclic substituents, and the branching of the alkyl radical at the endocyclic nitrogen atom. Research on methods to obtain polymer isoindigo thin films and the use of additives will, in our opinion, significantly improve the efficiency of materials. In addition, the first work on combining polyaromatic acceptor and heterocyclic donor fragments in one macromolecule on the isoindigo platform showed the possibility of designing one-component nonfullerene solar cells. The high stability of polymeric isoindigo in air, at elevated temperature, under redox conditions, under laser irradiation, and the biocompatibility make it possible to conclude that the design of these compounds is promising for mass spectrometry, for catalysts for hydrogen production, and for photothermal cancer therapy.

## References

[1] Xiao Z, Hao Y, Liu B, Qian L (2002). Leuk Lymphoma.

[2] Huang M, Lin H-S, Lee Y S, Ho P C (2014). Int J Oncol.

[3] Bogdanov A V, Musin L I, Mironov V F (2015). ARKIVOC.

[4] Stalder R, Mei J, Reynolds J R (2010). Macromolecules.

[5] Mei J, Graham K R, Stalder R, Reynolds J R (2010). Org Lett.

[6] Deng P, Zhang Q (2014). Polym Chem.

[7] Tan S E, Sarjadi M S (2017). Polym Sci, Ser B.

[8] Kini G P, Jeon S J, Moon D K (2020). Adv Mater (Weinheim, Ger).

[9] Wang Y, Yu Y, Liao H, Zhou Y, McCulloch I, Yue W (2020). Acc Chem Res.

[10] Wang L, Bai S, Wu Y, Liu Y, Yao J, Fu H (2020). Angew Chem, Int Ed.

[11] Eichhorn S H, El‐Ballouli A O, Cassar A, Kaafarani B R (2021). ChemPlusChem.

[12] Hadsadee S, Promarak V, Sudyoadsuk T, Keawin T, Kungwan N, Jungsuttiwong S (2020). J Electron Mater.

[13] Liu X, Ye L, Zhao W, Zhang S, Li S, Su G M, Wang C, Ade H, Hou J (2017). Mater Chem Front.

[14] Ganguly A, Zhu J, Kelly T L (2017). J Phys Chem C.

[15] Hu J, Xu K, Shen L, Wu Q, He G, Wang J-Y, Pei J, Xia J, Sfeir M Y (2018). Nat Commun.

[16] Wen Z, Wu J I-C (2020). Chem Commun.

[17] Jung J W (2017). Dyes Pigm.

[18] Shaker M, Lee J-H, Park B, Lee S, Lee K, Lee J-S (2020). Synth Met.

[19] Yu D, Liu Y, Fan Q, Xiao M, Tan H, Wang Y, Yang R, Zhu W (2016). Tetrahedron.

[20] Dinçalp H, Saltan G M, Zafer C, Mutlu A (2018). Spectrochim Acta, Part A.

[21] Zhou H, Sun X, Zhang Z, Yu Y, Huang M, Zhao B (2018). Dyes Pigm.

[22] Miao J, Meng B, Liu J, Wang L (2018). Chem Commun.

[23] Cho H-H, Han G, Younts R, Lee W, Gautam B R, Lee S, Lee C, Kim T, Kim F S, Gundogdu K (2017). J Mater Chem A.

[24] Liang L, Chen X-Q, Xiang X, Ling J, Shao W, Lu Z, Li J, Wang W, Li W-S (2017). Org Electron.

[25] Shaker M, El-Hendawy M M, Park B, Lee K (2019). New J Chem.

[26] Li M, Zhang G, Xiong L, Zhu M, Pei Y, Peng Q, Liu Y (2018). Dyes Pigm.

[27] Ji C, Yin L, Xie B, Wang X, Li X, Zhang J-J, Ni J, Li Y (2016). Synth Met.

[28] Lee J-Y, Lee S-M, Lee S-H, Kim D-H, Lee S H, Lee Y-S (2016). Chem Phys Lett.

[29] Chen X, Zhang Z, Liu J, Wang L (2017). Polym Chem.

[30] Miao J, Xu H, Meng B, Liu J, Wang L (2018). Chin J Chem.

[31] Deng P, Lei Y, Wu B, Zheng X, Lu Y, Zhu F, Ong B S (2016). Dyes Pigm.

[32] Kim J, Park S Y, Han G, Chae S, Song S, Shim J Y, Bae E, Kim I, Kim H J, Kim J Y (2016). Polymer.

[33] Mori H, Hara S, Nishinaga S, Nishihara Y (2017). Macromolecules.

[34] Park G E, Choi S, Shin J, Cho M J, Choi D H (2016). Org Electron.

[35] Jung E H, Ahn H, Jo W H, Jo J W, Jung J W (2019). Dyes Pigm.

[36] Zhou D, Doumon N Y, Abdu-Aguye M, Bartesaghi D, Loi M A, Koster L J A, Chiechi R C, Hummelen J C (2017). RSC Adv.

[37] Grand C, Zajaczkowski W, Deb N, Lo C K, Hernandez J L, Bucknall D G, Müllen K, Pisula W, Reynolds J R (2017). ACS Appl Mater Interfaces.

[38] Chang C-Y, Huang Y-C, Tsao C-S, Chen C-A, Su C-J, Su W-F (2017). Phys Chem Chem Phys.

[39] Tegegne N A, Abdissa Z, Mammo W, Uchiyama T, Okada-Shudo Y, Galeotti F, Porzio W, Andersson M R, Schlettwein D, Vohra V (2020). J Phys Chem C.

[40] Liao S-F, Chen C-T, Chao C-Y (2017). ACS Macro Lett.

[41] Tegegne N A, Wendimu H, Abdissa Z, Mammo W, Andersson M R, Hone F G, Andoshee D M, Olaoye O, Bosman G (2020). J Mater Sci: Mater Electron.

[42] Bini K, Xu X, Andersson M R, Wang E (2018). Macromol Chem Phys.

[43] Liu S, Firdaus Y, Thomas S, Kan Z, Cruciani F, Lopatin S, Bredas J-L, Beaujuge P M (2018). Angew Chem, Int Ed.

[44] Schroeder B C, Chiu Y-C, Gu X, Zhou Y, Xu J, Lopez J, Lu C, Toney M F, Bao Z (2016). Adv Electron Mater.

[45] Gu K L, Zhou Y, Gu X, Yan H, Diao Y, Kurosawa T, Ganapathysubramanian B, Toney M F, Bao Z (2017). Org Electron.

[46] Neophytou M, Bryant D, Lopatin S, Chen H, Hallani R K, Cater L, McCulloch I, Yue W (2018). Macromol Rapid Commun.

[47] Zhu L, Jiang C, Chen G, Zhou Z, Li Q (2017). Org Electron.

[48] Seri M, Gedefaw D, Prosa M, Tessarolo M, Bolognesi M, Muccini M, Andersson M R (2017). J Polym Sci, Part A: Polym Chem.

[49] Jeon S J, Lee T H, Han Y W, Moon D K (2018). Polymer.

[50] Sun X, Zhang Z, Hou R, Huang M, Zhao B, Tan S (2017). Dyes Pigm.

[51] Cong Z, Liu H, Wang W, Liu J, Zhao B, Guo Z, Gao C, An Z (2017). Dyes Pigm.

[52] Kwon N Y, Kang H, Park S H, Kim H J, Kim C Y, Park S, Cho M J, Choi D H (2020). Dyes Pigm.

[53] Wang X, Cheng C, Li Y, Wang F (2018). Polymers (Basel, Switz).

[54] Yu C, Xu Y, Li C, Feng G, Yang F, Li J, Li W (2018). Chin J Chem.

[55] Zhang B, An N, Wu H, Geng Y, Sun Y, Ma Z, Li W, Guo Q, Zhou E (2020). Sci China: Chem.

[56] Li Z, Xu X, Zhang W, Genene Z, Mammo W, Yartsev A, Andersson M R, Janssen R A J, Wang E (2017). J Mater Chem A.

[57] Wang X, Lv L, Gu W, Wang X, Dong T, Yang Z, Cao H, Huang H (2017). Dyes Pigm.

[58] Peng W, Tan H, Xiao M, Chen J, Tao Q, Duan X, Wang Y, Liu Y, Yang R, Zhu W (2016). Eur Polym J.

[59] Gao X, Zhao Z (2015). Sci China: Chem.

[60] Yuvaraja S, Nawaz A, Liu Q, Dubal D, Surya S G, Salama K N, Sonar P (2020). Chem Soc Rev.

[61] Yang J, Zhao Z, Wang S, Guo Y, Liu Y (2018). Chem.

[62] Shinamura S, Osaka I, Miyazaki E, Takimiya K (2011). Heterocycles.

[63] Larik F A, Faisal M, Saeed A, Abbas Q, Kazi M A, Abbas N, Thebo A A, Khan D M, Channar P A (2018). J Mater Sci: Mater Electron.

[64] Quinn J T E, Zhu J, Li X, Wang J, Li Y (2017). J Mater Chem C.

[65] Liu Y, Wang F, Chen J, Wang X, Lu H, Qiu L, Zhang G (2018). Macromolecules.

[66] You L, Chaudhry S T, Zhao Y, Liu J, Zhao X, He J, Mei J (2017). Polym Chem.

[67] Ashizawa M, Zheng Y, Tran H, Bao Z (2020). Prog Polym Sci.

[68] Zhu X, Zhang S-R, Zhou Y, Han S-T (2021). Polym Int.

[69] Ashizawa M, Masuda N, Higashino T, Kadoya T, Kawamoto T, Matsumoto H, Mori T (2016). Org Electron.

[70] Shaker M, Park B, Lee S, Lee K (2020). Dyes Pigm.

[71] Trinh C K, Lee H-J, Choi J W, Shaker M, Kim W, Lee J-S (2018). New J Chem.

[72] Shaker M, Hayashi H, Yamada H (2021). Dyes Pigm.

[73] Qiao X, Wei Q, Wu H, Li H (2020). Macromol Chem Phys.

[74] Lin Y-C, Chen F-H, Chiang Y-C, Chueh C-C, Chen W-C (2019). ACS Appl Mater Interfaces.

[75] Xue G, Zhao X, Qu G, Xu T, Gumyusenge A, Zhang Z, Zhao Y, Diao Y, Li H, Mei J (2017). ACS Appl Mater Interfaces.

[76] Tran D T, Gumyusenge A, Luo X, Roders M, Yi Z, Ayzner A L, Mei J (2020). ACS Appl Polym Mater.

[77] Shih C-C, Lee W-Y, Lu C, Wu H-C, Chen W-C (2017). Adv Electron Mater.

[78] Li Q-Y, Yao Z-F, Lu Y, Zhang S, Ahmad Z, Wang J-Y, Gu X, Pei J (2020). Adv Electron Mater.

[79] Yang J, Zhao Z, Geng H, Cheng C, Chen J, Sun Y, Shi L, Yi Y, Shuai Z, Guo Y (2017). Adv Mater (Weinheim, Ger).

[80] Liu L, Du Y, Ge F, Wang X, Zhang G, Lu H, Qiu L (2018). Appl Phys Lett.

[81] Dharmapurikar S S, Arulkashmir A, Mahale R Y, Chini M K (2017). J Appl Polym Sci.

[82] Lee J, Shin E-S, Kim Y-J, Noh Y-Y, Yang C (2020). J Mater Chem C.

[83] Wen H-F, Wu H-C, Aimi J, Hung C-C, Chiang Y-C, Kuo C-C, Chen W-C (2017). Macromolecules.

[84] Zhang G, Dai Y, Liu Y, Liu J, Lu H, Qiu L, Cho K (2017). Polym Chem.

[85] Yen H-C, Lin Y-C, Chen W-C (2021). Macromolecules.

[86] Ding Y, Jiang L, Du Y, Kim S, Wang X, Lu H, Zhang G, Cho K, Qiu L (2020). Chem Commun.

[87] Shin E-S, Ha Y H, Gann E, Lee Y-J, Kwon S-K, McNeill C R, Noh Y-Y, Kim Y-H (2018). ACS Appl Mater Interfaces.

[88] Gao Y, Deng Y, Tian H, Zhang J, Yan D, Geng Y, Wang F (2017). Adv Mater (Weinheim, Ger).

[89] Shi K, Zhang W, Liu X, Zou Y, Yu G (2017). Polymer.

[90] Wei C, Tang Z, Zhang W, Huang J, Zhou Y, Wang L, Yu G (2020). Polym Chem.

[91] Park W-T, Kim G, Yang C, Liu C, Noh Y-Y (2016). Adv Funct Mater.

[92] Liu Q, Bottle S E, Sonar P (2020). Adv Mater (Weinheim, Ger).

[93] Du Y, Ding Y, Ge F, Wang X, Ma S, Lu H, Zhang G, Qiu L (2019). Dyes Pigm.

[94] Bao W W, Li R, Dai Z C, Tang J, Shi X, Geng J T, Deng Z F, Hua J (2020). Front Chem (Lausanne, Switz).

[95] Shaker M, Park B, Lee J-H, Kim W, Trinh C K, Lee H-J, Choi J w, Kim H, Lee K, Lee J-S (2017). RSC Adv.

[96] Dharmapurikar S S, Chithiravel S, Mane M V, Deshmukh G, Krishnamoorthy K (2018). Chem Phys Lett.

[97] Onwubiko A, Yue W, Jellett C, Xiao M, Chen H-Y, Ravva M K, Hanifi D A, Knall A-C, Purushothaman B, Nikolka M (2018). Nat Commun.

[98] Zhang H, Zhao Z, Zhao N, Xie Y, Cai M, Wang X, Liu Y, Lan Z, Wan X (2017). RSC Adv.

[99] Zhao N, Ai N, Cai M, Wang X, Pei J, Wan X (2016). Polym Chem.

[100] Ganguly A, He K, Hendsbee A D, Abdelsamie M, Bennett R N, Li Y, Toney M F, Kelly T L (2020). ACS Appl Mater Interfaces.

[101] Bennett R N, Hendsbee A D, Ngai J H L, Ganguly A, Li Y, Kelly T L (2020). ACS Appl Electron Mater.

[102] Song H, Deng Y, Jiang Y, Tian H, Geng Y (2018). Chem Commun.

[103] Huang Y, Chen H, Yang J, Tian W, Wang W (2017). Polym Chem.

[104] Jo J W, Kim J H, Jung J W (2016). Dyes Pigm.

[105] Wu H-C, Hong C-W, Chen W-C (2016). Polym Chem.

[106] Lu C-F, Shih C-W, Chen C-A, Chin A, Su W-F (2018). Adv Funct Mater.

[107] Vu D L, Lin T-F, Lin T-H, Wu M-C (2020). Polymers (Basel, Switz).

[108] Zhou C, Zhao J, Ye J, Tange M, Zhang X, Xu W, Zhang K, Okazaki T, Cui Z (2016). Carbon.

[109] Soylemez S, Goker S, Toppare L (2019). New J Chem.

[110] Lu Q, Zhang X, Cai W, Wang Y, Yang C, Chen Y, Zhang W, Zhang Z, Niu H, Wang W (2019). Sol Energy Mater Sol Cells.

[111] Lu Q, Cai W, Zhang X, Yang C, Ge H, Chen Y, Niu H, Wang W (2018). Eur Polym J.

[112] Gu H, Ming S, Lin K, Chen S, Liu X, Lu B, Xu J (2018). Electrochim Acta.

[113] Leem G, Black H T, Shan B, Bantang J P O, Meyer T J, Reynolds J R, Schanze K S (2018). ACS Appl Energy Mater.

[114] Horatz K, Giampà M, Karpov Y, Sahre K, Bednarz H, Kiriy A, Voit B, Niehaus K, Hadjichristidis N, Michels D L (2018). J Am Chem Soc.

[115] Mery A, Bernard P, Valero A, Alper J P, Herlin-Boime N, Haon C, Duclairoir F, Sadki S (2019). J Power Sources.

[116] Jiang Y, Zheng X, Deng Y, Tian H, Ding J, Xie Z, Geng Y, Wang F (2018). Angew Chem, Int Ed.

[117] Yang T, Liu L, Deng Y, Guo Z, Zhang G, Ge Z, Ke H, Chen H (2017). Adv Mater (Weinheim, Ger).

[118] Dong T, Wen K, Chen J, Xie J, Fan W, Ma H, Yang L, Wu X, Xu F, Peng A (2018). Adv Funct Mater.

[119] Chang K, Liu Y, Hu D, Qi Q, Gao D, Wang Y, Li D, Zhang X, Zheng H, Sheng Z (2018). ACS Appl Mater Interfaces.

[120] Jiang Y, Duan X, Bai J, Tian H, Ding D, Geng Y (2020). Biomaterials.

[121] Shao W, Yang C, Li F, Wu J, Wang N, Ding Q, Gao J, Ling D (2020). Nano-Micro Lett.

